# Occurrence, Distribution, and Genetic Diversity of Alfalfa (*Medicago sativa* L.) Viruses in Four Major Alfalfa-Producing Provinces of China

**DOI:** 10.3389/fmicb.2021.771361

**Published:** 2022-01-13

**Authors:** Zhipeng Guo, Tingting Zhang, Zhao Chen, Junpeng Niu, Xuewen Cui, Yue Mao, Mahmood Ul Hassan, Hafiz Abdul Kareem, Nan Xu, Xin Sui, Shuanghong Gao, Momi Roy, Jian Cui, Quanzhen Wang

**Affiliations:** ^1^Department of Grassland Science, College of Grassland Agriculture, Northwest A&F University, Yangling, China; ^2^Key Laboratory of Animal Genetics, Breeding and Reproduction of Shaanxi Province, College of Animal Science and Technology, Northwest A&F University, Yangling, China; ^3^Department of Plant Science, College of Life Sciences, Northwest A&F University, Yangling, China

**Keywords:** alfalfa, viruses, high-throughput sequencing, small RNA, incidence, genetic diversity, China

## Abstract

Alfalfa (*Medicago sativa* L.) is one of the most widely cultivated forage crops in the world. China is the second largest producer of alfalfa in terms of the planting area worldwide, with Gansu, Henan, Inner Mongolia, and Shaanxi provinces being the production hubs. Alfalfa viruses have been reported on a small-scale survey in some of these areas, but they have not been well characterized. In the present study, seven viruses were detected in 12 fields of 10 cities/counties of the four abovementioned provinces by high-throughput sequencing and assembly of small RNA. Their incidence, distribution, and genetic diversity were analyzed by enzyme-linked immunosorbent assay, polymerase chain reaction (PCR)/reverse transcription-PCR and clone sequencing. The results showed that alfalfa mosaic virus (AMV), pea streak virus (PeSV), lucerne transient streak virus (LTSV), alfalfa dwarf virus (ADV), Medicago sativa alphapartitivirus 1 (MsAPV1), MsAPV2, and alfalfa leaf curl virus (ALCV) were the main viruses infecting alfalfa in four examined provinces. AMV and MsAPV1 had the highest incidences in all 4 provinces. SDT analysis of the 7 viruses isolated in China revealed a highly conserved among AMV, LTSV, ADV, MsAPV1, MsAPV2, and ALCV, but the sequence was a high variation between China isolates to abroad isolates in PeSV, ADV, and ALCV. To our knowledge, this is the first report of ADV in Inner Mongolia and Gansu, ALCV in Inner Mongolia, MsAPV1 and MsAPV2 in all 4 provinces, and PeSV and LTSV in China. These findings provide a basis for future research on the genetic evolution of alfalfa viruses in China and on strategies to prevent diseases in alfalfa caused by these viruses.

## Introduction

Alfalfa (*Medicago sativa* L.), which is referred to as “the queen of forages,” is the most important perennial leguminous forage crop worldwide owing to its high nutritional value and value for feeding livestock (Samac et al., 2015). The area of alfalfa cultivation is approximately 32 million hectares globally and is increasing with the development of the animal husbandry market (Samac et al., 2015). In China, alfalfa was planted on about 4.72 million hectares in 2015 (Guo et al., 2020). The major alfalfa-producing provinces include Gansu, Inner Mongolia, Xinjiang, Ningxia, Heilongjiang, Hebei, Shaanxi, Sichuan and Henan, which together produce more than 85% of the total alfalfa production in the country. Gansu and Shaanxi are the leading alfalfa producers in Northwestern China, while the Inner Mongolia Autonomous Region and Henan are the major producers in Northern and Central China, respectively.

To date, about 47 alfalfa viruses have been reported worldwide (Supplementary Table 1); of these, 11 have been previously reported in China including alfalfa mosaic virus (AMV; Guo et al., 2019; Li et al., 2021), alfalfa dwarf virus (ADV; Xuehelati et al., 2020), alfalfa leaf curl virus (ALCV; Guo et al., 2020), tomato mosaic virus (ToMV; Wen and Nan, 2015), cowpea mosaic virus (CPMV), bean yellow mosaic virus (BYMV), white clover mosaic virus (WCMV; Zhou et al., 2016), bean leafroll virus (BLRV; Li et al., 2019), Medicago sativa deltapartitivirus 1 (MsDPV1), Medicago sativa amalgavirus 1 (MsAV1; Li et al., 2021), and Medicago sativa alphapartitivirus 1 (MsAPV1; Kim et al., 2018; Nemchinov et al., 2018b; Li et al., 2021). The main symptoms caused by these viruses are macular mosaicism, mottling, ringspot, reddening, etiolation, shrinkage, mosaic shrinkage, and dwarfism (Guo et al., 2020; Samarfard et al., 2020).

High-throughput sequencing (HTS)—which can detect ultra-low levels of plant viruses—and bioinformatics analysis are important tools for the discovery of novel DNA or RNA viruses infecting plants (Mokili et al., 2012). About 10 alfalfa viruses have been identified by HTS technology (Bejerman et al., 2015, 2016; Nemchinov et al., 2017, 2018a; Raza et al., 2017; Kim et al., 2018; Gaafar et al., 2019; Samarfard et al., 2020).

Identifying viruses and analyzing their prevalence and distribution in alfalfa fields are critical for the development of effective disease management strategies. However, very few studies have been conducted in China on alfalfa virus diseases and most were small-scale investigations. In this study we conducted a large-scale survey of viruses in 12 alfalfa fields of 10 cities/counties in Gansu, Henan, Inner Mongolia Autonomous Region, and Shaanxi provinces of China and characterized their prevalence and molecular variability of alfalfa viruses by small (s)RNA HTS, PCR and RT-PCR.

## Materials and Methods

### Plant Material

**FIGURE 1:**
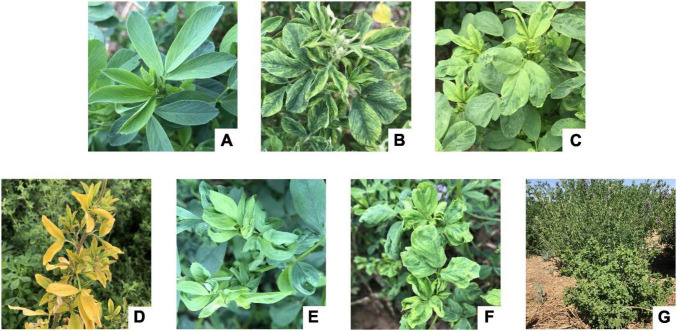
Symptom types of alfalfa virus disease in the field. **(A)** Health; **(B)** macular mosaicism; **(C)** mottling; **(D)** etiolation; **(E)** shrinkage; **(F)** mosaic shrinkage; **(G)** dwarfism.

**FIGURE 2:**
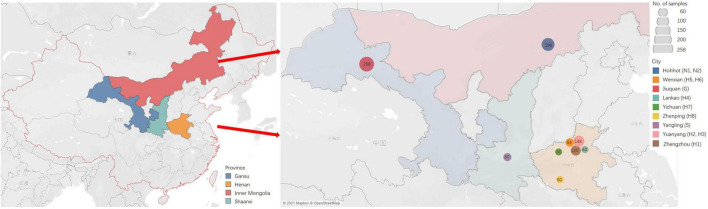
The sites of samples with virus-induced-symptoms in Inner Mongolia, Gansu, Shaanxi and Henan provinces of China.

Tender leaves showing virus-like symptoms, including macular mosaicism (*n* = 104), mottling (*n* = 138), etiolation (*n* = 52), shrinkage (*n* = 328), mosaic shrinkage (*n* = 350), and dwarfism (*n* = 96) (Figure 1 and Supplementary Table 2) were collected in May and June 2020 from alfalfa plants growing in Gansu, Henan, Inner Mongolia Autonomous Region, and Shaanxi provinces (Figure 2). The 1,068 samples were collected and included 258 from Jiuquan city, Gansu (G; N39°37′, E98°47′); 80 from Helinger county, Inner Mongolia Autonomous Region (N1; N40°39′, E111°59′); 118 from Tumote Left Banner, Inner Mongolia Autonomous Region (N2; N40°35′, E111°46′); 80 from Yangling district, Xianyang city, Shaanxi (S; N34°18′, E108°0.06′); 120 from Zhengzhou city, Henan (H1; N34°54′, E113°45′); 108 from Yuanyang county-1, Henan (H2; N35°06′, E113°57′); 40 from Yuanyang county-2, Henan (H3; N35°01′, E113°41′); 60 from Lankao county, Henan (H4; N34°49′, E114°59′); 44 from Wenxian county-1, Henan (H5; N34°53′, E113°09′); 40 from Wenxian county-2, Henan (H6; N34°51′, E113°07′); 60 from Yichuan county, Henan (H7; N34°18′, E112°22′); and 60 from Zhenping county, Henan (H8; N32°58′, E112°13′). The samples in each jurisdiction were collected from one field. All samples were immediately transported to the laboratory on dry ice and stored at −80°C until use.

### RNA Isolation and Deep Sequencing

Total RNA was extracted from each one of the 1,068 samples using TRIzol reagent (Zhonghuihecai, Shaanxi, China) according to the manufacturer’s instructions. For HTS, total RNA was extracted from a pooled sample including 6 leaves exhibiting each of the above-mentioned virus-like symptoms from each location, respectively. And 12 RNA samples from 12 locations were, respectively, subjected to single-end sRNA HTS to detect viruses using the Illumina Hiseq4000 platform at Biomarker Technologies (Beijing, China) and the Illumina Nextseq550 platform at Sangon Biotech (Shanghai, China). The sRNA libraries for deep sequencing were constructed as previously described (Lu et al., 2007). Raw reads were filtered and cleaned by removing those of low quality, with a proportion of unknown bases > 10%, or without adapter sequences along with insert fragments, adapter sequences, and reads > 34 nt and < 15 nt. The Bowtie v1.0.0 software (Langmead et al., 2009) was used to remove rRNA, tRNA, small nuclear RNA, small nucleolar RNA, non-coding RNA and repetitive sequences from the 15- to 34-nt clean reads for alignment to the Silva (Pruesse et al., 2007), GtRNAdb (Chan and Lowe, 2009), Rfam (Griffiths-Jones et al., 2003), Repbase (Jurka et al., 2005) databases. The remaining clean reads were assembled and spliced using SPAdes software (K-mer value = 17; Bankevich et al., 2012). The assembled contigs were compared with GenBank Virus RefSeq nucleotide and protein databases and NCBI Non-redundant protein and nucleotide sequences databases using BLASTn and BLASTx (1e-5).

### Virus Detection by PCR and Reverse Transcription (RT)-PCR

To detect viruses in alfalfa samples, total DNA and RNA was extracted from each of the 1,068 samples using the Plant Genomic DNA Extraction Kit (Beijing Solarbio Science and Technology Co., Beijing, China) and the TRIzol reagent (Zhonghuihecai, Shaanxi, China), respectively, according to the manufacturer’s recommendations. For RNA samples, the cDNA was synthesized using HiScript II reverse transcriptase and oligo (dT)23VN primer (Vazyme Biotech, Nanjing, China) according to the manufacturer’s instructions. PCR (used for DNA virus detection) and RT-PCR (used for RNA virus detection) were performed using the Taq PCR Master Mix Kit (Jiangsu CoWin Biosciences, Taizhou, China) with the following programs: predenaturation at 94°C for 2 min; 35 cycles of denaturation at 94°C for 30 s, annealing at 56°C for 30 s, and extension at 72°C for 2 min; and final extension at 72°C for 2 min. Primers used to amplify specific sequences of AMV, pea streak virus (PeSV), lucerne transient streak virus (LTSV), ADV, MsAPV1, Medicago sativa alphapartitivirus 2 (MsAPV2), and ALCV are listed in Supplementary Table 3. The PCR products were resolved by gel electrophoresis on a 1.2% agarose gels and purified using SanPrep Column DNA Gel Extraction Kit (Sangon Biotech). The purified PCR products were verified by Sanger sequencing by Sangon Biotech.

### Cloning and Sequencing Analysis

The purified PCR products consisting of the sequence fragments of virus capsid protein were cloned into the pUCm-T vector (Sangon Biotech) and transformed into *Escherichia coli* DH5α competent cells (Sangon Biotech). Positive clones were verified by PCR and at least 3 independently derived clones were sequenced by Sangon Biotech. By cloning and sequencing, we obtained the sequences of the complete coat protein (CP) gene sequences of PeSV, LTSV, MsAPV1, and MsAPV2, and the complete Nucleocapsid (N) gene of ADV, and the complete genome sequence of ALCV. Almost the whole genomic sequence was obtained by splicing the assembled contigs sequences and cloning sequencing sequences with DNAMAN v6 (Lynnon Biosoft, QC, Canada) software (Supplementary Tables 4–10).

Viral reference sequences were downloaded from GenBank (Supplementary Tables 4–10) and aligned using Muscle with default parameters (Kumar et al., 2016). Phylogenetic and molecular evolutionary analyses were performed using MEGA v7.0 (Kumar et al., 2016) with 1,000 bootstrap replicates. A phylogenetic tree was constructed by the maximum likelihood (ML) method using MEGA v7.0 software. The other parameters were as follows: substitution type = nucleotide; model/method = Jukes–Cantor model; rates among sites = uniform; gaps/missing data treatment = complete deletion; ML = heuristic (nearest neighbor interchange); initial tree for ML = automatically constructed (maximum parsimony).

Pairwise sequence alignment of viral reference sequences (Supplementary Tables 4–10) was performed using SDT software (Muhire et al., 2014). The parameters were as follows: alignment programs = Muscle; Cluster sequences using a neighbor joining tree.

Recombination analysis was performed by SimPlot 3.5 software (Lole et al., 1999). The nucleotide sequence of the isolate from different host and country were used as the reference sequence (Supplementary Tables 4–10), then Similarity plot and Bootscanning analysis were performed using SimPlot 3.5 (Lole et al., 1999). Genetic Algorithm Recombination Detection (GARD) was used to detect the Recombination sites and evaluate their reliability (Pond et al., 2006a,b).

### AMV, PeSV, LTSV, and ALCV Detection by Enzyme-Linked Immunosorbent Assay

Five samples of each virus (AMV, PeSV, LTSV, and ALCV)-positive responses to RT-PCR were used for enzyme-linked immunosorbent assay (ELISA), respectively. AMV, PeSV, LTSV, and ALCV using ELISA kits obtained from Shanghai Yuanxin Biotechnology Co., Ltd., to identify the virus following the procedure provided by the supplier.

## Results

### Detection of Viruses Infecting Alfalfa by sRNA High-Throughput Sequencing

Each RNA sample was extracted from a pooled sample of 6 leaves exhibiting each of the above-mentioned virus-like symptoms from each location and pooled, fragmented into libraries, and sequenced on an Illumina platform (San Diego, CA, United States), respectively. From the 12 HTS data, 6,933,449–15,668,658 clean reads were selected from 9,788,810–16,082,935 raw reads (Supplementary Table 11); 23–111 contigs were mapped to 22 viral reference sequences (Supplementary Table 12). The contigs of sample G were aligned to nucleotide sequences of AMV, PeSV, ADV, MsAPV1, BLRV, and raspberry vein chlorosis virus. The contigs of sample N1 were aligned to nucleotide sequences of AMV, PeSV, LTSV, ADV, MsAPV1, ALCV, BLRV, alfalfa latent virus (ALV), Allium fistulosum carlavirus, Ilex cornuta carlavirus, garlic common latent virus, birch carlavirus, cowpea mild mottle virus, cherry twisted leaf-associated virus, and cherry green ring mottle virus. The contigs of sample N2 were aligned to nucleotide sequences of AMV, PeSV, ADV, ALCV, BLRV, and ALV. The contigs of sample S were aligned to nucleotide sequences of AMV, MsAPV1, and MsAPV2. The contigs of sample H1 were aligned to nucleotide sequences of AMV, ADV, MsAPV1, ALCV, grapevine cabernet sauvignon reovirus, raspberry latent virus, and cassava frogskin virus. The contigs of sample H2 were aligned to nucleotide sequences of AMV, ADV, MsAPV1, and ALCV. The contigs of sample H3 were aligned to nucleotide sequences of AMV, MsAPV1, MsAPV2, ALCV, and MsAV1. The contigs of sample H4 were aligned to nucleotide sequences of AMV, MsAPV1, and ALCV. The contigs of sample H5 were aligned to nucleotide sequences of AMV, ADV, MsAPV1, and ALCV. The contigs of sample H6 were aligned to nucleotide sequences of AMV and ALCV. The contigs of sample H7 were aligned to nucleotide sequences of AMV, MsAPV1, and ALCV. The contigs of sample H8 were aligned to nucleotide sequences of AMV, MsAPV1, and capsicum chlorosis virus (Supplementary Table 12). Nearly complete genomic sequences of PeSV, ALCV, and AMV were assembled from samples G, H1, and H8, respectively (Supplementary Table 12). Those assembly sequences were derived from each mixed sample; thus, could potentially be chimeric. Hence a further molecular validation should be performed by PCR and RT-PCR.

### Detection of Viruses Infecting Alfalfa Detected by PCR and RT-PCR

**FIGURE 3:**
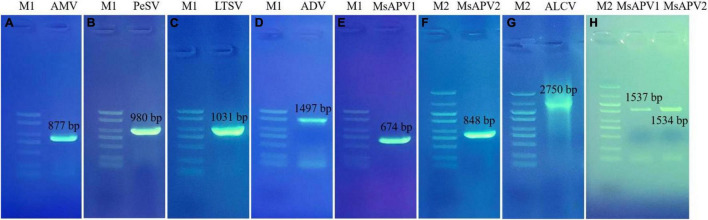
PCR validation for the presence of seven viruses. **(A)** AMV; **(B)** PeSV; **(C)** LTSV; **(D)** ADV; **(E)** MsAPV1; **(F)** MsAPV2; **(G)** ALCV, **(H)** MsAPV1 (left) and MsAPV2 (right). M1 and M2 represent DL 2000 DNA marker and DL 5000 DNA marker, respectively. The specific bands in A-H were amplified by corresponding primers from Supplementary Table 3, respectively.

Twenty-five primer pairs were used to detect 22 different viruses by PCR and RT-PCR. Fifteen of the viruses tested negative, but 7 of the viruses (AMV, PeSV, LTSV, ADV, MsAPV1, MsAPV2, and ALCV) tested positive in alfalfa pooled samples. After that, we detected the above seven viruses in 1,068 samples, separately. Of these, AMV, MsAPV1, and MsAPV2 were detected in all 4 provinces; PeSV was detected in Gansu and Inner Mongolia; LTSV was detected in Inner Mongolia; ADV was detected in Gansu, Henan, and Inner Mongolia, and ALCV was detected in Henan and Inner Mongolia. The PCR products of ALCV and RT-PCR products of AMV, PeSV, LTSV, ADV, MsAPV1, and MsAPV2 showed distinct bands by agarose gel electrophoresis, with sizes of 2,750, 877, 980, 1031, 1497, 674 bp (MsAPV1); 848 bp (MsAPV2); 1,537 bp (MsAPV1 CP gene); and 1,534 bp (MsAPV1 CP gene) (Figure 3), confirming the presence of the 7 viruses in the alfalfa samples.

### AMV, PeSV, LTSV, and ALCV Detection by Enzyme-Linked Immunosorbent Assay

**FIGURE 4:**
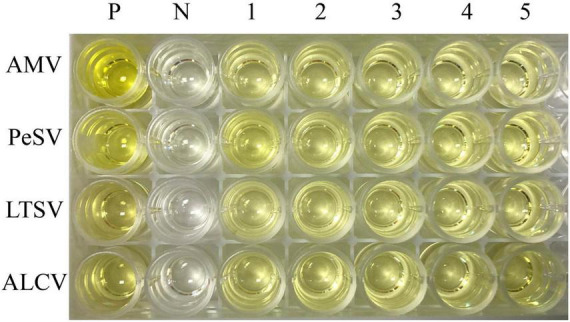
The results of ELISA for AMV, PeSV, LTSV, and ALCV detection. P: Positive control (*Medicago sativa* leaves that were infected by AMV, PeSV, LTSV, and ALCV, respectively), N: Negative control (virus-free alfalfa leaves), 1–5: Five samples of each virus that were detected positive for RT-PCR were also detected positive by ELISA. Line 1–4 represent AMV, PeSV, LTSV, and ALCV, respectively.

The results of ELISA for four kinds of viruses (AMV, PeSV, LTSV, and ALCV) detection were shown in Figure 4. As shown in Figure 4, five samples of each virus that were detected positive for the above viruses by RT-PCR were also detected positive by ELISA, respectively. While the negative control of alfalfa without showing symptoms typical of virus infection were not detected for these four kinds of viruses by ELISA.

### Virus Prevalence and Distribution

**FIGURE 5:**
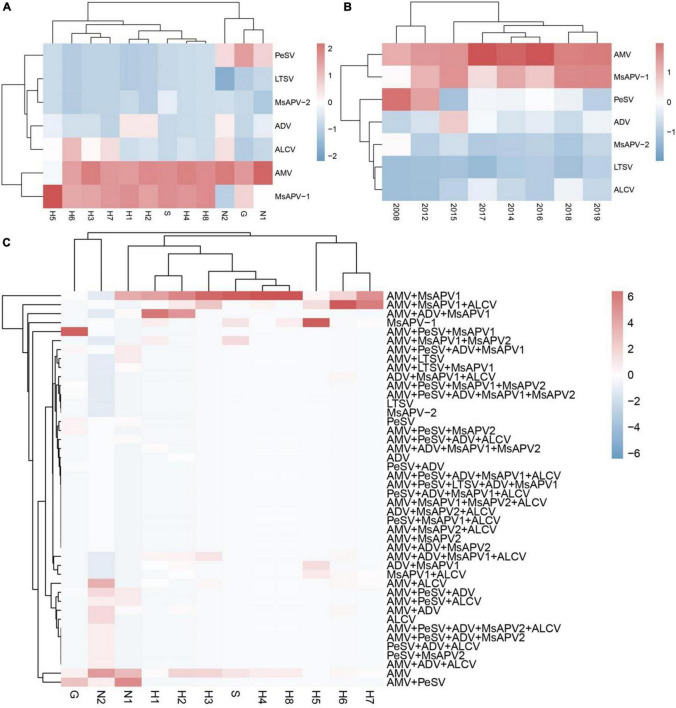
The colored heat map of detection rate of AMV, PeSV, LTSV, ADV, MsAPV1, MsAPV2 and ALCV of samples, in different locations **(A)**, planted in different year **(B)**, and incidence of various viruses with both single and multiple infection in different alfalfa-growing locations **(C)**. Jiuquan (G), Helinger (N1), Tumote Left Banner (N2), Yangling (S), Zhengzhou (H1), Yuanyang-1 (H2), Yuanyang-2 (H3), Lankao (H4), Wenxian-1 (H5), Wenxian-2 (H6), Yichuan (H7) and Zhenping (H8). The red color represents a high detection rate of virus, while the blue color represents a low detection rate of virus.

The viruses had the highest prevalence among samples from all surveyed cities/counties in Henan province (100%), followed by Tumote Left Banner, Inner Mongolia Autonomous Region (89.83%); Jiuquan, Gansu (84.50%); Helinger county, Inner Mongolia Autonomous Region (80.00%); and Yangling, Shaanxi (65.00%) (Supplementary Table 13). AMV was detected at the highest rate, followed by MsAPV1, PeSV, ADV, ALCV, MsAPV2, and LTSV. AMV and MsAPV1 were detected in all 12 alfalfa-growing regions of the 4 provinces (Figure 5A). The main symptoms of samples single-infected with AMV were etiolation (10/52) and macular mosaicism (14/104), while samples only infected with MsAPV1 showed shrinkage (18/328) and mosaic shrinkage (18/350) as the major symptoms (Supplementary Table 2). In samples infected with multiple viruses, the most frequent combinations were AMV + MsAPV1, AMV + PeSV, AMV + PeSV + MsAPV1, AMV + ADV + MsAPV1, and AMV + MsAPV1 + ALCV (Figure 5C). The main symptoms of samples infected with virus combinations were as follows: AMV + MsAPV1, shrinkage (94/328), macular mosaicism (28/104), and mosaic shrinkage (86/350); AMV + PeSV, macular mosaicism (12/104) and etiolation (4/52); AMV + PeSV + MsAPV1, mottling (26/138) and shrinkage (40/328); AMV + ADV + MsAPV1, dwarfism (18/96) and mosaic shrinkage (36/350); and AMV + MsAPV1 + ALCV, dwarfism (12/96), mosaic shrinkage (28/350), and shrinkage (26/328) (Supplementary Table 2). The detection rates of AMV, MsAPV1, ADV, ALCV, and LTSV were lower in samples planted before 2012 than in those planted after 2012. On the contrary, PeSV and MsAPV2 detection rates were higher among samples planted before 2012 than among those planted after 2012 (Figure 5B).

**FIGURE 6:**
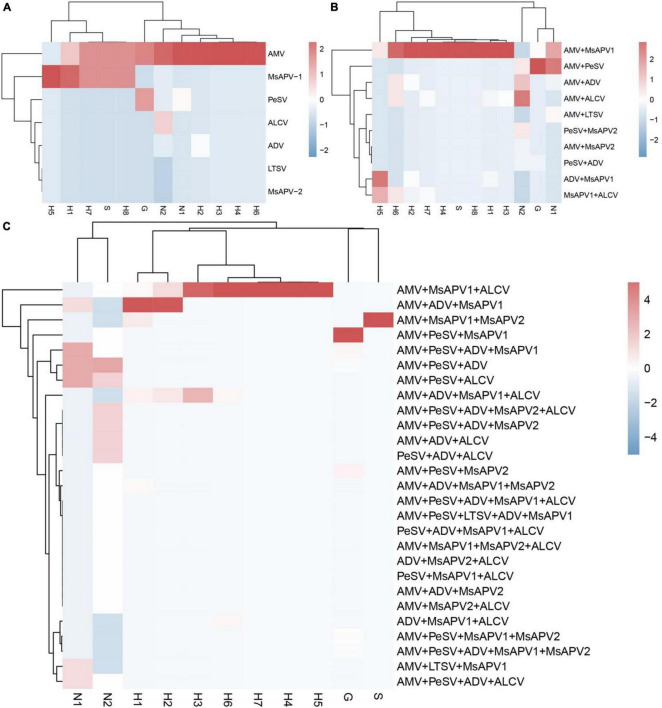
The colored heat map of Incidence of single infection **(A)**, dual infection **(B)** and multiple infection **(C)** in different alfalfa-growing locations. Jiuquan (G), Helinger (N1), Tumote Left Banner (N2), Yangling (S), Zhengzhou (H1), Yuanyang-1 (H2), Yuanyang-2 (H3), Lankao (H4), Wenxian-1 (H5), Wenxian-2 (H6), Yichuan (H7) and Zhenping (H8). The red color represents a high detection rate of virus, while the blue color represents a low detection rate of virus.

The incidences of single and multiple infections varied across 12 alfalfa-growing locations in the 4 provinces (Supplementary Tables 14–25). AMV and MsAPV1 were main viruses involved in single infections in all of the fields (Supplementary Tables 14–25). The sites with the highest single-infection rates were Yuanyang-2, Yuanyang-1, Helinger, Tumote Left Banner, Lankao and Zhenping (AMV); and Wenxian-1, Zhenping, Yangling, and Zhengzhou (MsAPV1) (Figure 6A). The most common 2-virus combinations were AMV + MsAPV1 and AMV + PeSV; the sites with the highest dual infection rates were Lankao, Zhenping, Yuanyang-2, Yangling, Yichuan, Yuanyang-1 and Zhengzhou (AMV + MsAPV1); and Helinger and Jiuquan (AMV + PeSV) (Figure 6B). The most frequent combinations of multiple viruses were AMV + PeSV + MsAPV1, AMV + ADV + MsAPV1, and AMV + MsAPV1 + ALCV; the sites with the highest multiple infection rates were Jiuquan (AMV + PeSV + MsAPV1); Zhengzhou and Yuanyang-1 (AMV + ADV + MsAPV1); and Wenxian-2 and Yichuan (AMV + MsAPV1 + ALCV) (Figure 6C).

### Recombination Analysis of Alfalfa Viruses CP Gene or N Gene

**FIGURE 7:**
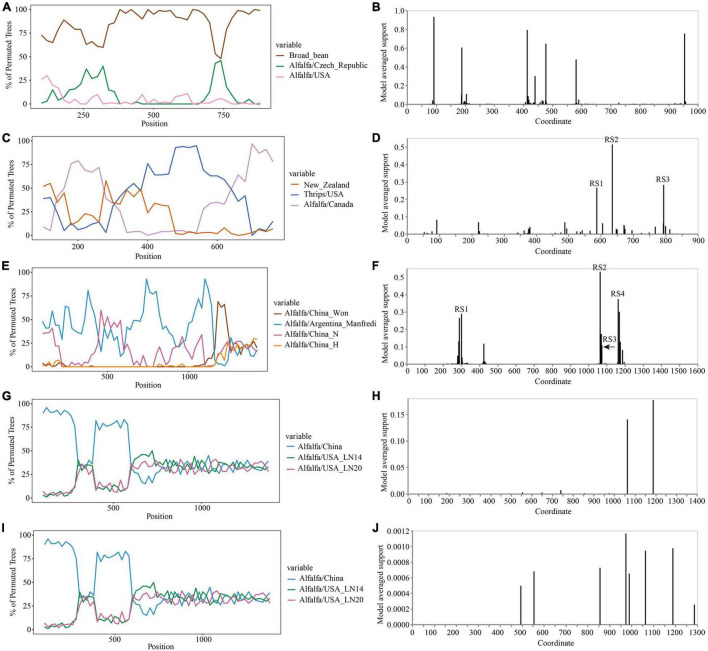
Recombination analysis of CP gene (PeSV, LTSV, MsAPV1, and MsAPV2) and N gene (ADV). Bootscanning validation of recombinant isolates [**(A)** PeSV; **(C)** LTSV; **(E)** ADV; **(G)** MsAPV1; and **(I)** MsAPV2]. The nucleotide sequence of the isolate from different host and country were used as the reference sequence, and the nucleotide sequence of the isolate from 4 provinces in this study was as the test sequence. GARD detection of recombinant sites of alfalfa viruses [**(B)** PeSV; **(D)** LTSV; **(F)** ADV; **(H)** MsAPV1; and **(J)** MsAPV2]. RS represents the recombination site.

The Simplot analysis and GARD found no recombination evidence of MsAPV1, and MsAPV2 (Figures 7G–J). Simplot analysis did not detect recombination signals in PeSV isolates (Figure 7A), but GARD found evidence of recombination with up to 8 breakpoints (Figure 7B). On the contrary, recombination signals of LTSV and ADV isolates from 4 provinces were detected by using Simplot (Figures 7C,E), and the recombination sites of these isolates were further confirmed by GARD (Figures 7D,F). For LTSV, GARD found 3 recombination sites, which were located at 587, 635, and 794 sites of CP gene, respectively (Figure 7D), with an average model approval rate of 26.41%, 51.17%, and 28.58%, respectively (Figure 7D). For ADV, GARD found 4 recombination sites, which were located at 286 (approval rate 30.80%), 1,064 (46.51%), 1,073 (8.94%), and 1,163 (42.08%) positions of the N gene, respectively (Figure 7F).

### Recombination Analysis of Whole Genome Sequence of Alfalfa Viruses

**FIGURE 8:**
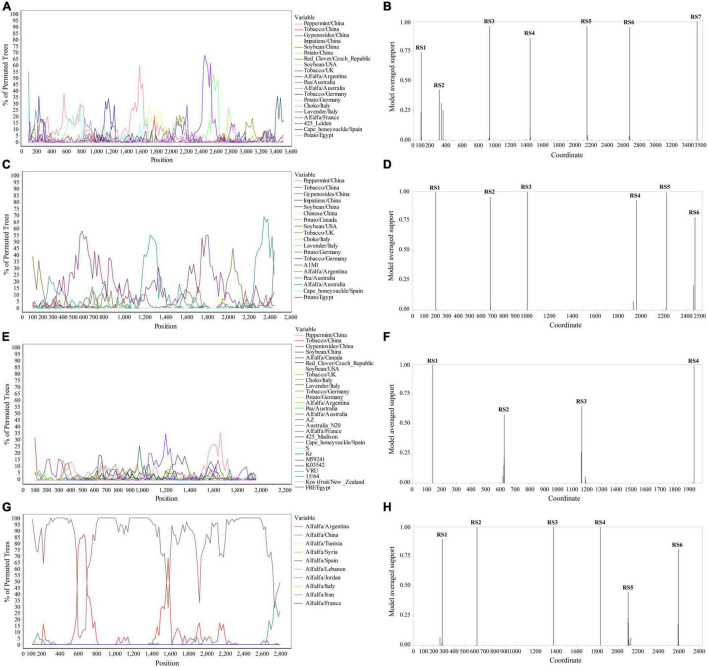
Recombination analysis of whole genome sequence (AMV and ALCV). Bootscanning validation of recombinant isolates [**(A)** AMV-RNA1; **(C)** AMV-RNA2; **(E)** AMV-RNA3; and **(G)** ALCV]. The nucleotide sequence of the isolate from different host and country were used as the reference sequence, and the nucleotide sequence of the isolate from 4 provinces in this study was as the test sequence. GARD detection of recombinant sites of alfalfa viruses [**(B)** AMV-RNA1; **(D)** AMV-RNA2; **(F)** AMV-RNA3; and **(H)** ALCV]. RS represents the recombination site.

The Simplot analysis and GARD found recombination evidence of AMV, and ALCV (Figure 8). Simplot analysis detected recombination signals in AMV and ALCV isolates (Figures 8A,C,E,G), and GARD found evidence of recombination with up to 7, 6, 4, and 6 breakpoints in AMV-RNA1, AMV-RNA2, AMV-RNA3, and ALCV, respectively (Figures 8B,D,F,H). For AMV-RNA1, GARD found 7 recombination sites, which were located at 99, 321, 942, 1,441, 2,142, 2,670, and 3,500 sites of AMV-RNA1 genome, respectively (Figure 8B), with an average model approval rate of 74.23, 42.53, 95.98, 86.04, 95.72, 95.42, and 99.99%, respectively (Figure 8B). For AMV-RNA2, six recombination sites were detected at 202 (99.48%), 681 (95.47%), 1,004 (99.98%), 1,961 (92.51%), 2,225 (99.08%), and 2,474 (78.41%) positions of the AMV-RNA2 genome, respectively (Figure 8D). For AMV-RNA3, four recombination sites were located at 138 (99.70%), 631 (57.69%), 1,165 (64.67%), and 1,939 (98.76%) positions of the AMV-RNA3 genome, respectively (Figure 8F). For ALCV, GARD found 6 recombination sites, which were detected at 285 (89.69%), 625 (99.51%), 1,373 (99.99%), 1,832 (99.75%), 2,104 (44.81%), and 2,595 (81.17%) positions of the ALCV genomic sequence, respectively (Figure 8H).

### Evolution Analysis of Alfalfa Viruses

**FIGURE 9:**
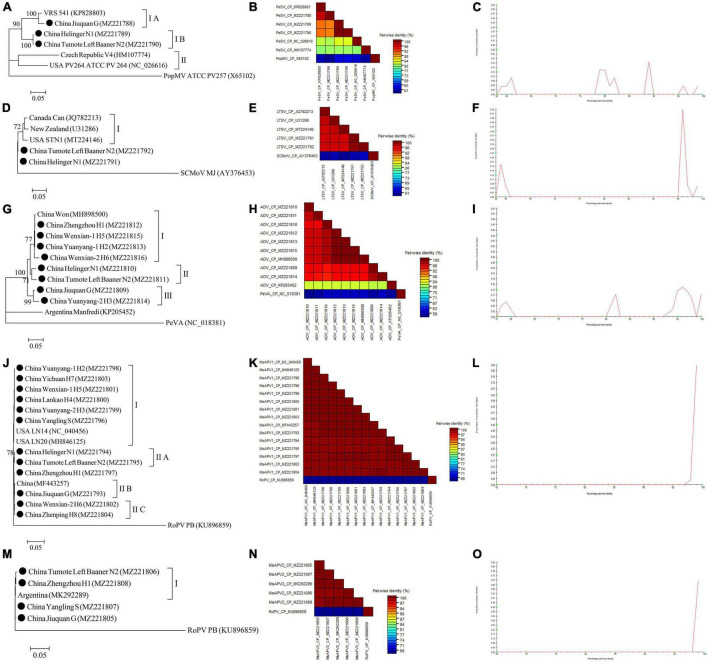
Rooted Maximum Likelihood phylogenetic trees basing on complete CP gene nucleotide sequences [**(A)** PeSV; **(D)** LTSV; **(J)** MsAPV1, and **(M)** MsAPV2), and complete N gene nucleotide sequences [**(G)** ADV] respectively. The black dot marks the sequence obtained in this study. Jiuquan denote location in Gansu province, China. Helinger and Tumote Left Banner denote location in Inner Mongolia Autonomous Region, China. Yangling denote location in Shaanxi province, China. Zhengzhou, Yuanyang-1, Yuanyang-2, Lankao, Wenxian-1, Wenxian-2, Yichuan and Zhenping denote location in Henan province, China. Bootstrap values below 70% were not shown. The SDT interface **(B,C,E,F,H,I,K,L,N,O)**: Color-coded pairwise identity matrix generated from PeSV sequences **(B)**, LTSV sequences **(E)**, ADV sequences **(H)**, MsAPV1 sequences **(K)**, and MsAPV2 sequences **(N)**. Each colored cell represents a percentage identity score between two sequences (one indicated horizontally to the left and the other vertically at the bottom). Pairwise identity frequency distribution plot of PeSV **(C)**, LTSV **(F)**, ADV **(I)**, MsAPV1 **(L)**, and MsAPV2 **(O)**. The horizontal axis indicates percentage pairwise identities, and the vertical axis indicates proportions of these identities within the distribution.

The complete CP gene sequences of 4 alfalfa viruses (PeSV, LTSV, MsAPV1, and MsAPV2) and the complete N gene sequences of ADV identified in this study were deposited in GenBank (Supplementary Tables 5–9). For PeSV, three complete PeSV CP gene sequences and 1 complete poplar mosaic virus (PopMV, as outgroup sequence) CP gene sequence downloaded from GenBank, and 3 complete PeSV CP gene sequences in this study (Supplementary Table 5) were used to build a phylogenetic tree (Figure 9A). The phylogenetic tree of 7 complete CP gene sequences showed that PeSV isolates formed 2 groups (Figure 9A). The Jiuquan G isolate was clustered in group IA and was most closely related to isolate VRS541. Helinger N1 and Tumote Left Banner N2 isolates were placed in group IB, while the PopMV ATCC PV257 isolate was an outgroup (Figure 9A). There was significant variation between the PeSV isolates from China and those from other countries (Figure 9B), and the nucleotide identity between these isolates was from 79.8 to 100.0% (Figure 9C).

For LTSV, three complete LTSV CP gene sequences and 1 complete subterranean clover mottle virus (SCMoV, as outgroup sequence) CP gene sequence downloaded from GenBank, and 2 complete LTSV CP gene sequences in this study (Supplementary Table 6) were used to build a phylogenetic tree (Figure 9D). The phylogenetic tree of the 6 complete CP sequences revealed that the Canada, New Zealand, and United States isolates of LTSV were clustered in group I (Figure 9D). The isolates from Helinger and Tumote Left Banner of Inner Mongolia Autonomous Region were outliers, while the SCMoV MJ isolate was an outgroup (Figure 9D). There was low variation between the LTSV isolates from China and those from other countries (Figure 9E), and the nucleotide identity between these isolates was higher than 96.5% (Figure 9F).

For ADV, two complete ADV N gene sequences and 1 complete persimmon virus A (PeVA, as outgroup sequence) N gene sequences downloaded from GenBank, and 8 complete ADV N gene sequences in this study (Supplementary Table 7) were used to build a phylogenetic tree (Figure 9G). The phylogenetic tree of the 11 complete N gene sequences showed that ADV isolates were divided into 3 groups (Figure 9G). Zhengzhou H1, Wenxian-1 H5, Yuanyang-1 H2, and Wenxian-2 H6 clustered together in group I and showed the closest relationship to the Won isolate identified in China. Isolates Helinger N1 and Tumote Left Banner N2 were placed in group II. Isolates Jiuquan G and Yuanyang-2 H3 were clustered in group III. The Manfredi Isolate from Argentina was an outlier, while PeVA was an outgroup (Figure 9G). All the ADV isolates from China showed a high degree of homogeneity (Figure 9H) with > 93.4% nucleotide identity between these isolates (Figure 9I), but a significant variation between China isolates and Argentina isolates (Figure 9H) and the nucleotide identity of isolates from both countries was as low as 81.2–81.8% (Figure 9I).

For MsAPV1, three complete MsAPV1 CP gene sequences and 1 complete rose partitivirus (RoPV, as outgroup sequence) CP gene sequence downloaded from GenBank, and 12 complete MsAPV1 CP gene sequences in this study (Supplementary Table 8) were used to build a phylogenetic tree (Figure 9J). The phylogenetic tree of the 16 complete CP sequences showed that MsAPV1 isolates were divided into 2 groups (Figure 9J). The Yuanyang-1 H2, Yichuan H7, Wenxian-1 H5, Lankao H4, Yuanyang-2 H3 isolates from Henan, and Yangling S from Shaanxi, and LN20, and LN14 from United States were clustered in group I. The Helinger N1 and Tumote Left Banner N2 isolate from Inner Mongolia were clustered in group IIA. The isolate Jiuquan G, which was most closely related to an isolate from China, was placed in group IIB. The Wenxian-2 H6 and Zhenping H8 isolates were placed in group IIC, while the PoPV PB isolate was an outgroup (Figure 9J). There was a highly conserved between the MsAPV1 isolates in this study (Figure 9K) with > 99.4% nucleotide identity between these isolates (Figure 9L).

For MsAPV2, one complete MsAPV2 CP gene sequence and 1 complete RoPV CP gene sequence (as outgroup sequence) downloaded from GenBank, and 4 complete MsAPV2 CP gene sequences in this study (Supplementary Table 9) were used to build a phylogenetic tree (Figure 9M). The phylogenetic tree of the 6 complete CP sequences showed that the Tumote Left Banner N2 and Zhengzhou H1 isolates, which were most closely related to the isolate from Argentina, clustered together in group I (Figure 9M). The isolates Yangling S and Jiuquan G were outliers, while the PoPV PB isolate was an outgroup (Figure 9M). All the MsAPV2 isolates in this study showed a high degree of homogeneity (Figure 9N) with > 99.5% nucleotide identity between these isolates (Figure 9O).

**FIGURE 10:**
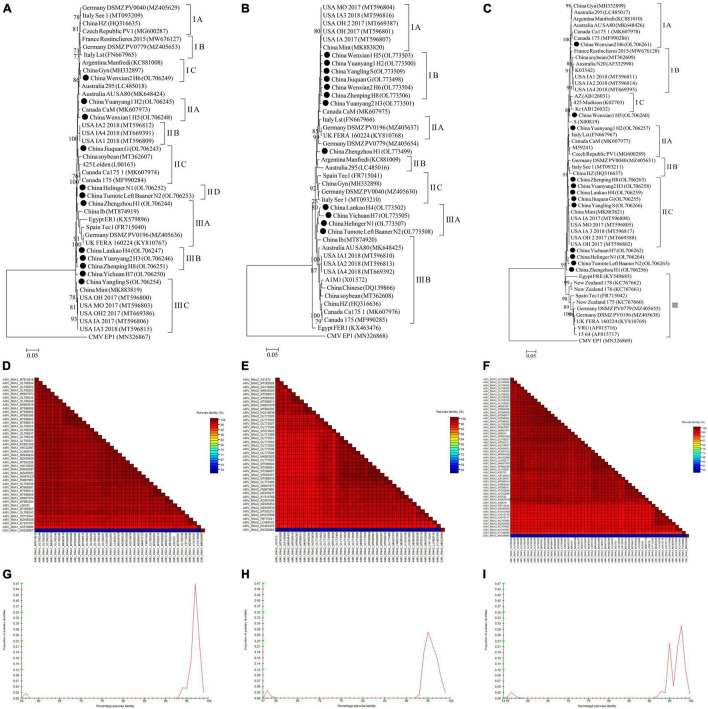
Rooted Maximum Likelihood phylogenetic trees basing on complete AMV-RNA nucleotide sequences of AMV-RNA1 **(A)**, AMV-RNA2 **(B)**, and AMV-RNA3 **(C)** respectively. The black dot marks the sequence obtained in this study. Jiuquan denote location in Gansu province, China. Helinger and Tumote Left Banner denote location in Inner Mongolia Autonomous Region, China. Yangling denote location in Shaanxi province, China. Zhengzhou, Yuanyang-1, Yuanyang-2, Lankao, Wenxian-1, Wenxian-2, Yichuan and Zhenping denote location in Henan province, China. Bootstrap values below 70% were not shown. The SDT interface **(D–F,G–I)**: Color-coded pairwise identity matrix generated from AMV-RNA1 sequences **(D)**, AMV-RNA2 sequences **(E)** and AMV-RNA3 sequences **(F)**. Each colored cell represents a percentage identity score between two sequences (one indicated horizontally to the left and the other vertically at the bottom). Pairwise identity frequency distribution plot of AMV-RNA1 **(G)**, AMV-RNA2 **(H)** and AMV-RNA3 **(I)**. The horizontal axis indicates percentage pairwise identities, and the vertical axis indicates proportions of these identities within the distribution.

For AMV, almost whole genomic sequence of AMV identified in this study were deposited in GenBank (Supplementary Table 4). For AMV-RNA1, thirty-one AMV-RNA1 sequences and 1 complete cucumber mosaic virus (CMV, as outgroup sequence) RNA1 sequence downloaded from GenBank, and 12 AMV-RNA1 sequences in this study (Supplementary Table 4) were used to build a phylogenetic tree (Figure 10A). The phylogenetic tree of 44 RNA1 sequences showed that the isolates were divided into 3 groups (Figure 10A). Isolates Wenxian-2 H6 was placed in group IC and was most closely related to the isolate Gyn from China. The isolates Yuanyang-1 H2 and Wenxian-1 H5 clustered together in group IIA and were most closely related to the isolate CaM from Canada. Isolates Jiuquan G was placed in group IIC and was most closely related to the isolate from China. The isolates Helinger N1 and Tumote Left Banner N2 were clustered in group IID. Isolate Zhengzhou H1 was placed in group IIIA and was most closely related to isolate Ib from China. The isolates Lankao H4, Yuanyang-2 H3, and Zhenping H8 clustered in group IIIB. Yangling S isolate was placed in group IIIC and was most closely related to isolate Mint from China, while the CMV EP15 isolate was an outgroup (Figure 10A). All the AMV isolates in this study showed a high degree of homogeneity (Figure 10D) with > 94.3% nucleotide identity between these isolates (Figure 10G).

For AMV-RNA2, twenty-nine AMV-RNA2 sequences and 1 complete cucumber mosaic virus (CMV, as outgroup sequence) RNA2 sequence downloaded from GenBank, and 12 AMV-RNA2 sequences in this study (Supplementary Table 4) were used to build a phylogenetic tree (Figure 10B). The phylogenetic tree of 44 RNA2 sequences showed that the isolates were divided into 3 groups (Figure 10B). Isolates Wenxian-1 H5, Yuanyang-1 H2, Yangling S, Jiuquan G, Wenxian-2 H6, and Zhenping H8 were placed in group IB and were most closely related to the isolate Mint from China. Lankao H4, Yichuan H7, Helinger N1, and Tumote Left Banner N2 isolates clustered together in group IIIA and were most closely related to the isolate Ib from China. Isolates FER1 from Egypt was an outlier, while the CMV EP1 isolate was an outgroup (Figure 10B). There was a highly conserved between the AMV isolates from China (Figure 10E) with > 96.15% nucleotide identity between these isolates (Figure 10H) and a low variation between the AMV isolates from China and those from other countries (Figure 10E), and the nucleotide identity between these isolates was higher than 93.6% (Figure 10H).

For AMV-RNA3, forty AMV-RNA3 sequences and 1 complete cucumber mosaic virus (CMV, as outgroup sequence) RNA3 sequence downloaded from GenBank, and 12 AMV-RNA3 sequences in this study (Supplementary Table 4) were used to build a phylogenetic tree (Figure 10C). The phylogenetic tree of 53 RNA3 sequences showed that the isolates were divided into 3 groups (Figure 10C). Isolates Wenxian-2 H6 was placed in group IA and was most closely related to the isolate from Canada. Yuanyang-1 H2 isolate was clustered in group IIA and was most closely related to the isolate Lst from Italy. Zhenping H8, Yuanyang-2 H3, Lankao H4, Jiuquan G, and Yangling S clustered together in group IIC and were most closely related to the isolate Mint from China, while the CMV EP1 isolate was an outgroup (Figure 10C). The AMV isolates in this study showed a high degree of homogeneity (Figure 10F) with > 97.3% nucleotide identity between these isolates (Figure 10I). All the AMV isolates showed a high degree of homogeneity (Figure 10F) which nucleotide identity was higher than 91.6% (Figure 10I).

**FIGURE 11:**
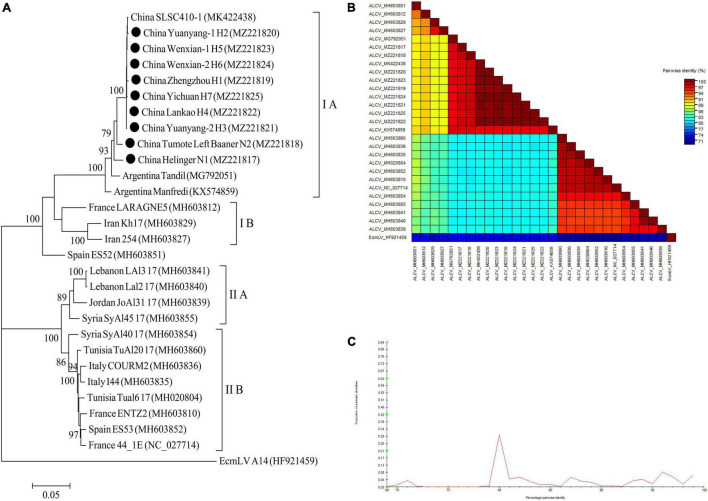
Rooted Maximum Likelihood phylogenetic trees basing on nucleotide sequences of ALCV whole genome sequence **(A)**. The black dot marks the sequence obtained in this study. Helinger and Tumote Left Banner denote location in Inner Mongolia Autonomous Region, China. Zhengzhou, Yuanyang-1, Yuanyang-2, Lankao, Wenxian-1, Wenxian-2 and Yichuan denote location in Henan province, China. Bootstrap values below 70% were not shown. The SDT interface **(B,C)**: Color-coded pairwise identity matrix generated from ALCV whole genome sequences **(B)**. Each colored cell represents a percentage identity score between two sequences (one indicated horizontally to the left and the other vertically at the bottom). Pairwise identity frequency distribution plot of ALCV whole genome sequences **(C)**. The horizontal axis indicates percentage pairwise identities, and the vertical axis indicates proportions of these identities within the distribution.

For ALCV, nineteen ALCV whole genome sequences and 1 Euphorbia caput-medusae latent virus (EcmLV, as outgroup sequence) downloaded from GenBank, and 9 ALCV whole genome sequences in this study (Supplementary Table 10) were used to build phylogenetic trees (Figure 11A). The phylogenetic trees of 29 complete genomic sequences revealed that the isolates formed 2 groups (Figure 11A). All isolates from China and Argentina were placed in one group and were closely related. All of the isolates from Henan province in this study were clustered in 1 group and showed the closest relationship to the isolate SLSC410-1 isolate detected in alfalfa from Henan. The EcmLV A14 isolate was an outgroup (Figure 11A). All the ALCV isolates from China showed highly conserved (Figure 11B) with > 97.2% nucleotide identity between these isolates (Figure 11C), but a significant variation between China isolates and abroad isolates (Figure 11B) and the nucleotide identity of isolates between China and other countries was from 80.3 to 97.6% (Figure 11C).

## Discussion

This is the first large-scale survey of alfalfa viruses in 4 provinces of China (Gansu, Henan, Inner Mongolia, and Shaanxi). Seven viruses—namely, AMV, PeSV, LTSV, ADV, MsAPV1, MsAPV2, and ALCV—were detected in alfalfa plant samples as individual virus infections or as dual or multiple infections. Samples with virus-induced symptoms were frequently found to be infected with multiple viruses (Figure 5C). The main symptoms of samples infected with AMV only were etiolation and macular mosaicism (Supplementary Table 2), while those of samples infected with MsAPV1 or ALCV only were shrinkage and mosaic shrinkage (Supplementary Table 2). These symptoms may serve as biological indicators for diagnosing alfalfa virus infection, although additional studies are needed to establish the typical symptoms triggered by a single virus.

Eleven alfalfa viruses have been detected in specific alfalfa-growing provinces of China including ADV and BLRV in Xinjiang (Li et al., 2019; Xuehelati et al., 2020); AMV in Beijing, Gansu, Henan, and Xinjiang (Wen and Nan, 2015; Zhang, 2016; Guo et al., 2019; Li et al., 2021); ALCV in Henan (Guo et al., 2020); ToMV, WCMV, CPMV, and BYMV in Gansu (Wen and Nan, 2015; Zhou et al., 2016); and MsAPV1 in Beijing (Kim et al., 2018; Li et al., 2021). We report here for the first time the infection of alfalfa with the following viruses in the surveyed provinces: PeSV in Gansu and Inner Mongolia; LTSV in Inner Mongolia; ADV in Gansu, Henan, and Inner Mongolia; MsAPV1 and MsAPV2 in Gansu, Henan, Inner Mongolia, and Shaanxi; and ALCV in Inner Mongolia. Importantly, the incidence of MsAPV1 (65.36%) was almost as high as that of AMV (79.96%) in China (Figure 5A). AMV and MsAPV1 were detected at similar rates in Yichuan and Zhenping (Henan; Figure 5A). On the other hand, the incidence of MsAPV1 was higher than that of AMV in Zhengzhou and Wenxian (Henan; Figure 5A). AMV and MsAPV1 are the main RNA viruses infecting alfalfa in Beijing (Li et al., 2021); our results indicate that MsAPV1 is also the predominant virus in Henan.

The rate of detection of AMV in the 12 locations surveyed in this study ranged from 18.18% in Wenxian-1 to 100% in Yuanyang-2 and Lankao regions. This is in accordance with rates reported in other countries; for example, in a survey carried out in the Saudi Arabia, AMV was detected in alfalfa at rates ranging from 41.9 to 82.5% (Abdalla et al., 2020). In this study, the incidences of AMV in alfalfa grown for more than 2 years in China is > 90% (Figure 5B). AMV—the most widespread virus species infecting alfalfa globally—can infect up to 80% of plants in an alfalfa stand more than 2 years old and 100% of plants in 3-year-old stands (Samac et al., 2015); it can also reduce the alfalfa yield by 9%–82%, plant height by 7%–57% (Guo et al., 2019), and crude protein content by 42.70% (Han et al., 2019). AMV has a wide host range that includes more than 700 species in over 70 families. Alfalfa is an important perennial host of AMV and a reservoir for AMV strains, which can be transmitted by aphids to other host crops such as pea, chickpea, and tomato (Samac et al., 2015). Our data suggest that AMV is a significant threat to alfalfa production in China.

High variability is one of the typical characteristics of RNA viruses, mainly due to the lack of correction function of RNA-dependent RNA polymerase (RdRp) or replicase. RNA viruses have a very high mutation rate (about 10^–4^ nucleotides per replication cycle), which is also an evolutionary strategy of RNA viruses (Malpica et al., 2002). The ability to transfer the hereditary information encased inside capsids—the protective proteinaceous shells that include the centers of infection particles (virions)—is unique to bona fide viruses and recognizes them from other sorts of egotistical hereditary elements such as transposons and plasmids (Krupovič and Bamford, 2009). Recombination can have a significant effect on the evolutionary process and is of intriguing in its own right (Pond et al., 2006a). GARD has not required a non-recombinant reference arrangement and recombination between sequences is also accommodated, which can be run in parallel on a cluster of computers, and so is well suited to screen for recombination in big datasets (Pond et al., 2006a). In this study, recombination signals of LTSV, ADV, AMV, and ALCV isolates from 4 provinces were detected by using Simplot (Figures 7C,E, 8A,C,E,G), and GARD found the recombination sites of LTSV located at 635 sites of CP gene with an average model approval rate of 51.17% (Figure 7D). The recombination sites of ADV, which were located at 1,064 (46.51%) position of the N gene, respectively (Figure 7F). Most the recombination sites of AMV and ALCV were higher than 80.00 (Figures 8B,D,F,H). These results indicated that the confidence of the recombinant site is high, and the variation of these viruses is mainly caused by base site mutation and gene recombination. And these two factors play an important role in the evolution of these viruses and are the main factors for the formation of new strains of alfalfa viruses. Analyzing the whole genome sequence of the virus can get more mutation sites and sufficient time, which can help us understand the evolutionary relationship of the virus more comprehensively.

Taxonomic classification approaches which are based on pairwise genomic identity measures are potentially highly automatable and are progressively popular with the International Committee on Taxonomy of Viruses (ICTV). SDT, a virus classification tool based on pairwise sequence alignment and identity calculation, can produce publication-quality color-coded distance matrices and pairwise identity plots to further assist the classification of sequences according to the taxonomic demarcation criteria approved by ICTV (Muhire et al., 2014). LTSV and AMV isolate from alfalfa plants in China showed a high degree of homogeneity (Figures 9E, 10D–F). AMV isolates from Gansu and Shaanxi were closely related, and both were distantly related to isolates from Inner Mongolia (Figures 10A–C). ALCV sequences from the 4 surveyed provinces were highly conserved (Figures 11B,C) and most closely related to the isolates from Argentina (Figure 11A), but a significant variation to the isolates from other countries (Figures 11B,C). The results suggested that ALCV isolates in China originated from a single ALCV isolate similar to what has been reported for ALCV in Argentina (Davoodi et al., 2018). These authors also suggested that the virus most likely originated in Iran (Davoodi et al., 2018). For PeSV and ADV, there was significant variation between the isolates from China and those from other countries (Figures 9B,H). Chinese isolates MsAPV1 and MsAPV2 showed minor variations in the CP gene sequences (Figures 9K,N). MsAPV1 was first identified through an analysis of a public transcriptome dataset (Kim et al., 2018). The complete genome sequences of MsAPV1 and MsAPV2 were obtained based on that of MsAPV (Bejerman et al., 2019). In our study, we readily detected the RNA-dependent RNA polymerase (RdRp) gene of MsAPV1 and MsAPV2 and confirmed that these are different virus species (Bejerman et al., 2019).

High-throughput sequencing is widely used for the detection of plant viruses as it allows a comprehensive, large-scale, and unbiased analysis of the genome (Villamor et al., 2019; Bejerman et al., 2020). In the present study, most of the fragments in the high-quality assembly were mapped to the viral nucleic acid sequences of 7 viruses with high homology, and the viruses were verified by PCR/RT-PCR, cloning, and sequencing. Notably, we did not detect any unknown viruses.

## Conclusion

This study identified the main virus species infecting alfalfa in Gansu, Henan, Inner Mongolia, and Shaanxi provinces of China and analyzed their incidence, distribution, and genetic diversity. To our knowledge, as the virus host is alfalfa, this is the first report of PeSV and LTSV in China; ADV in Gansu and Inner Mongolia; ALCV in Inner Mongolia; and MsAPV1 and MsAPV2 in all 4 surveyed provinces. The incidence of MsAPV1 was high and close to that of AMV in China. SDT analysis of the 7 viruses isolated in China revealed a highly conserved among AMV, LTSV, ADV, MsAPV1, MsAPV2, and ALCV, but the sequence was a high variation between China isolates to abroad isolates in PeSV, ADV, and ALCV. These results provide a basis for the studies on the genetic evolution of alfalfa viruses, particularly the 7 species identified in this work, and can guide the development of strategies for preventing diseases in alfalfa caused by these viruses.

## Data Availability Statement

The datasets presented in this study can be found in online repositories. The names of the repository/repositories and accession number(s) can be found below: NCBI SRA BioProject, accession no: PRJNA761453.

## Author Contributions

ZG and QW conceived the ideas designed the methodology. ZG and JN collected alfalfa samples. ZG, TZ, JN, XC, YM, MH, HK, NX, XS, SG, and MR conducted the experiments. ZG, TZ, and ZC analyzed the data and wrote the manuscript. ZC, MH, JC, and QW edited the language of the manuscript. QW supervised the project and provided the constructive suggestions for revisions. All authors contributed to the article and approved the submitted version.

## Conflict of Interest

The authors declare that the research was conducted in the absence of any commercial or financial relationships that could be construed as a potential conflict of interest.

## Publisher’s Note

All claims expressed in this article are solely those of the authors and do not necessarily represent those of their affiliated organizations, or those of the publisher, the editors and the reviewers. Any product that may be evaluated in this article, or claim that may be made by its manufacturer, is not guaranteed or endorsed by the publisher.
