# Supplementary material for: Occurrence, Distribution, and Genetic Diversity of Alfalfa (Medicago sativa L.) Viruses in Four Major Alfalfa-Producing Provinces of China
Source: Front Microbiol. 2022 Jan 13;12:771361. doi: 10.3389/fmicb.2021.771361 (PMC8793692; doi:10.3389/fmicb.2021.771361)
Supplement: Supplementary file 1 [file Data_Sheet_1.docx]

Supplementary Table 1. The information of 47 viruses infecting alfalfa.

| NO. | Viruses | Reference | NO. | Viruses | Reference |
| --- | --- | --- | --- | --- | --- |
| 1 | alfalfa mosaic virus | Guo et al. 2019 | 25 | cactus virus X | Jiang et al. 2019a |
| 2 | alfalfa leaf curl virus | Guo et al. 2020 | 26 | Zhuye pepper nepovirus | Jiang et al. 2019a |
| 3 | alfalfa dwarf virus | Bejerman et al. 2015 | 27 | cowpea mild mottle virus | Jiang et al. 2019a |
| 4 | cowpea mosaic virus | Zhou et al. 2016 | 28 | alfalfa latent virus | Jiang et al. 2019a |
| 5 | bean yellow mosaic virus | Zhou et al. 2016 | 29 | strawberry latent ringspot virus | Jiang et al. 2019a |
| 6 | white clover mosaic virus | Zhou et al. 2016 | 30 | sweet potato virus C | Jiang et al. 2019a |
| 7 | clover yellow vein virus | Shah et al. 2006 | 31 | sweet potato chlorotic fleck virus | Jiang et al. 2019a |
| 8 | red clover vein mosaic virus | Al-Shahwan et al. 2016 | 32 | sweet potato feathery mottle virus | Jiang et al. 2019a |
| 9 | bean leafroll virus | Samarfard et al. 2020 | 33 | cycas necrotic stunt virus | Jiang et al. 2019b |
| 10 | Medicago sativa alphapartitivirus 1 | Samarfard et al. 2020 | 34 | clover yellow mosaic virus | Pratt 1961 |
| 11 | Medicago sativa amalgavirus 1 | Samarfard et al. 2020 | 35 | red clover mottle virus | Hampton et al. 1978 |
| 12 | chickpea chlorosis Australia virus | Samarfard et al. 2020 | 36 | lucerne Australian latent virus | Forster et al. 1985 |
| 13 | alfalfa ringspot-associated virus | Samarfard et al. 2020 | 37 | lucerne Australian symptomless virus | Remah et al. 2010 |
| 14 | alfalfa enamovirus 1 | Bejerman et al. 2016 | 38 | beet western yellows virus | Johnstone and Duffus 1984 |
| 15 | Medicago sativa alphapartitivirus 2 | Bejerman et al. 2019 | 39 | lucerne transient streak virus | Al-Shahwan et al. 2017 |
| 16 | alfalfa-associated nucleorhabdovirus | Gaafar et al. 2019 | 40 | cucumber mosaic virus | Al-Shahwan et al. 2017 |
| 17 | peanut stunt virus | Nemchinov et al. 2017 | 41 | tobacco streak virus | Al-Shahwan et al. 2017 |
| 18 | alfalfa virus S | Nemchinov et al. 2017 | 42 | bean common mosaic virus | Al-Shahwan et al. 2017 |
| 19 | alfalfa virus F | Nemchinov et al. 2018a | 43 | watermelon mosaic virus | Grogan et al. 1959 |
| 20 | Medicago sativa maraﬁvirus 1 | Nemchinov et al. 2018a | 44 | alfalfa enation virus | Samac et al. 2015 |
| 21 | Medicago sativa deltapartitivirus 1 | Kim et al. 2018 | 45 | red clover necrotic mosaic virus | Lynes et al. 1981 |
| 22 | cnidium vein yellowing virus | Jiang et al. 2019a | 46 | sweet clover necrotic mosaic virus | Hiruki 1987 |
| 23 | lychnis mottle virus | Jiang et al. 2019a | 47 | tomato mosaic virus | Wen and Nan 2015 |
| 24 | pea streak virus | Jiang et al. 2019a |  |  |  |

Supplementary Table 2. Summary of virus infection in different symptomatic samples.

| Virus infectious | Macular mosaic | Mottle mosaic | Etiolation | Shrinkage | Mosaic shrinkage | Dwarfism |
| --- | --- | --- | --- | --- | --- | --- |
| AMV | 14 | 12 | 10 | 22 | 18 | 6 |
| PeSV | 2 | 2 | 2 | 6 | 4 | 0 |
| ADV | 2 | 2 | 0 | 0 | 0 | 0 |
| MsAPV1 | 4 | 6 | 2 | 18 | 18 | 2 |
| ALCV | 0 | 0 | 0 | 4 | 2 | 0 |
| AMV+PeSV | 12 | 4 | 4 | 20 | 20 | 4 |
| AMV+LTSV | 0 | 0 | 0 | 2 | 2 | 0 |
| AMV+ADV | 0 | 0 | 0 | 4 | 8 | 2 |
| AMV+MsAPV1 | 28 | 30 | 10 | 94 | 86 | 22 |
| AMV+MsAPV2 | 0 | 0 | 0 | 0 | 2 | 0 |
| AMV+ALCV | 4 | 0 | 0 | 2 | 10 | 2 |
| PeSV+ADV | 4 | 0 | 0 | 0 | 0 | 0 |
| PeSV+MsAPV2 | 2 | 0 | 0 | 2 | 0 | 0 |
| ADV+MsAPV1 | 0 | 0 | 0 | 8 | 4 | 0 |
| MsAPV1+ALCV | 0 | 0 | 2 | 4 | 4 | 0 |
| AMV+PeSV+ADV | 0 | 2 | 0 | 6 | 4 | 0 |
| AMV+PeSV+MsAPV1 | 6 | 26 | 4 | 40 | 32 | 2 |
| AMV+PeSV+MsAPV2 | 4 | 2 | 2 | 4 | 0 | 2 |
| AMV+PeSV+ALCV | 0 | 0 | 2 | 4 | 0 | 2 |
| AMV+LTSV+MsAPV1 | 2 | 0 | 0 | 0 | 0 | 0 |
| AMV+ADV+MsAPV1 | 0 | 2 | 2 | 24 | 36 | 18 |
| AMV+ADV+MsAPV2 | 0 | 0 | 0 | 2 | 0 | 0 |
| AMV+ADV+ALCV | 0 | 0 | 0 | 2 | 4 | 4 |
| AMV+MsAPV1+MsAPV2 | 0 | 6 | 2 | 4 | 0 | 4 |
| AMV+MsAPV1+ALCV | 2 | 6 | 2 | 26 | 28 | 12 |
| AMV+MsAPV2+ALCV | 0 | 0 | 0 | 0 | 2 | 0 |
| PeSV+ADV+ALCV | 0 | 0 | 0 | 2 | 2 | 0 |
| PeSV+MsAPV1+ALCV | 0 | 0 | 0 | 0 | 0 | 2 |
| ADV+MsAPV1+ALCV | 0 | 0 | 0 | 0 | 2 | 0 |
| ADV+MsAPV2+ALCV | 0 | 0 | 0 | 0 | 2 | 0 |
| AMV+PeSV+ADV+MsAPV1 | 4 | 2 | 0 | 2 | 6 | 2 |
| AMV+PeSV+ADV+MsAPV2 | 2 | 0 | 2 | 0 | 0 | 0 |
| AMV+PeSV+ADV+ALCV | 0 | 0 | 0 | 0 | 2 | 2 |
| AMV+PeSV+MsAPV1+MsAPV2 | 2 | 0 | 0 | 0 | 2 | 2 |
| AMV+ADV+MsAPV1+MsAPV2 | 0 | 0 | 0 | 0 | 4 | 2 |
| AMV+ADV+MsAPV1+ALCV | 0 | 2 | 0 | 4 | 10 | 2 |
| AMV+MsAPV1+MsAPV2+ALCV | 2 | 0 | 0 | 0 | 0 | 0 |
| PeSV+ADV+MsAPV1+ALCV | 0 | 0 | 0 | 0 | 2 | 0 |
| AMV+PeSV+LTSV+ADV+MsAPV1 | 0 | 0 | 0 | 0 | 0 | 2 |
| AMV+PeSV+ADV+MsAPV1+MsAPV2 | 0 | 2 | 0 | 0 | 0 | 0 |
| AMV+PeSV+ADV+MsAPV1+ALCV | 0 | 0 | 0 | 0 | 2 | 0 |
| AMV+PeSV+ADV+MsAPV2+ALCV | 0 | 0 | 2 | 0 | 2 | 0 |
| Total | 104 | 138 | 52 | 328 | 350 | 96 |

Note: Abbreviation for virus: alfalfa mosaic virus, AMV; pea streak virus, PeSV; lucerne transient streak virus, LTSV; alfalfa dwarf virus, ADV; Medicago sativa alphapartitivirus 1, MsAPV1; Medicago sativa alphapartitivirus 2, MsAPV2; alfalfa leaf curl virus, ALCV.

Supplementary Table 3. All the primers used in this study.

| Primers | Sequence (5’-3’) | Product size (bp) | Application | Reference |
| --- | --- | --- | --- | --- |
| AMV-CP-F | AACGGTGCGTATAGATGCCG | 877 | Virus detection and CP gene clone for AMV | Guo et al., 2019 |
| AMV-CP-R | TTAATCCACCCAGTGGAGGTCA |  |  |  |
| PeSV-CP-F | ATACGCAAAAACCTTGAAAGTGCAAG | 980 | Virus detection and CP gene clone for PeSV | This study |
| PeSV-CP-R | AGGTTTTACTAGCAACCGCACAC |  |  |  |
| LTSV-CP-F | GGATCGTTGCTATCTCCAAT | 1034 | Virus detection and CP gene clone for LTSV | Raza et al., 2017 |
| LTSV-CP-R | CGAAAACCATCATACGGCTA |  |  |  |
| ADV-N-F | CGTCCACGTCGGATTCCAG | 1497 | Virus detection and N gene clone for ADV | This study |
| ADV-N-R | TTGGTCGTCATTCAGTCACCACT |  |  |  |
| ADV-F | TTGAATGATGACGACTGTGT | 1065 | Virus detection for ADV | Samarfard et al., 2018 |
| ADV-R | CCTAGCTTTCTGATCTTCCC |  |  |  |
| MsAPV1-F | CTCAAGTGGTCATGATCTC | 674 | Virus detection for MsAPV1 | Bejerman et al., 2019 |
| MsAPV1-R | GAGTAATGAAAGGATAGCAC |  |  |  |
| MsAPV1-CP-F | ATTTTCTACTCCCCTCCCCCAC | 1537 | CP gene clone for MsAPV1 | This study |
| MsAPV1-CP-R | CAAACAAGAAGACATGAAAACCGGCAA |  |  |  |
| MsAPV2-F | AAGACTAACGACCATGGAC | 848 | Virus detection for MsAPV2 | Bejerman et al., 2019 |
| MsAPV2-R | GCTAATAGTTGTAGTTCGTC |  |  |  |
| MsAPV2-CP-F  MsAPV2-CP-R | ATTTTCTACTCCCCTCCCCCAC | 1534 | Virus detection and CP gene clone for MsAPV2 | This study |
|  | ACAAGAAGACATGAAAACCGGCAAG |  |  |  |
| ALCV-F | CCCTGGCCTGCTAAAGTGGCCCAATTCAACATGG | 2750 | Virus detection and complete genome sequence clone for ALCV | Guo et al., 2020 |
| ALCV-R | CCAGGGGGCCTTATTCCTCTGGGACCG |  |  |  |
| BLRV-F | GAAGATCAAGCCAGGTTCA | 389 | Virus detection for BLRV | Ortiz et al., 2005 |
| BLRV-R | TCCAGCAATCTTGGCATCTC |  |  |  |
| RVCV-F  RVCV-R | TCYCGACATGATTGCTCATAC | 298 | Virus detection for RVCV | Jones et al., 2019 |
|  | GGGAACATCCTCATTATCTTY |  |  |  |
| ALV-F | CGATTGTGTCTCTGGTCATCTC | 684 | Virus detection for ALV | Nemchinov 2017 |
| ALV-R | ATAAAGATGGCAGAGCAACAGA |  |  |  |
| AFCLV-F | AGCAGAGCAGTTAATGATGTCGGTATC | 343 | Virus detection for AFCLV | This study |
| AFCLV-R | ACACTTCAGCCAAGCACTCTTCAG |  |  |  |
| ICCLV-F | ACGCCATTGAGATAAGTTACGCCTATG | 314 | Virus detection for ICCLV | This study |
| ICCLV-R | ACGGATACAACTGCTCTTACCACAAC |  |  |  |
| GCLV-F | ATGTCAGTGAGTGAAACAGAGG | 960 | Virus detection for GCLV | Parrano et al., 2012 |
| GCLV-R | CTAGTCTGCATTGTTGGATCC |  |  |  |
| BCLV-F | CCTCTTATCTGGTCTCGCTCTTTGTG | 207 | Virus detection for BCLV | This study |
| BCLV-R | CTCTCCGCCTTACTCCTCCTATCAC |  |  |  |
| CPMMV-F | CTGGCCAAGTGGTTTGTTTT | 1865 | Virus detection for CPMMV | Wei et al., 2020 |
| CPMMV-R | AACCGGGTTTACAATCCACA |  |  |  |
| CTLaV-F | TTGTTGTTTGCTGCTGTTGGAGTTTC | 286 | Virus detection for CTLaV | This study |
| CTLaV-R | GATGCTGAATGCGACTAGACCTACTG |  |  |  |
| CGRMV-F | CCTCATTCACATAGCTTAGGTTT | 948 | Virus detection for CGRMV | Wang et al., 2009 |
| CGRMV-R | ACTTTAGCTTCGCCCCGTG |  |  |  |
| GCSV-F | AGAGAGGGGTTGCACAAACTG | 719 | Virus detection for GCSV | Fan et al., 2021 |
| GCSV-R | CCAGCATTGTTAACTCCAGCC |  |  |  |
| RpLV-F | CACCGCCCAACCACAACTTCT | 547 | Virus detection for RpLV | Quito-Avila et al., 2011 |
| RpLV-R | CCTCGTGCTCGCTCTCCTTCATA |  |  |  |
| CFSV-F | TGGCCGGGAGAACAATAATA | 958 | Virus detection for CFSV | Calvert et al., 2008 |
| CFSV-R | GCGAAGTAAGTTCCGTCGTT |  |  |  |
| MsAGV1-F | GTTAGAACGCAAGCACGAACTTCAAG | 366 | Virus detection for MsAGV1 | This study |
| MsAGV1-R | TTCTTCACACGACAAGCATCAGGAC |  |  |  |
| CaCV-F | ATGTCTAMCGTYAGGCAAC | 800 | Virus detection for CaCV | Basavaraj et al., 2020 |
| CaCV-R | TYACACYTCWATAGAWGTACTAG |  |  |  |

Note: Abbreviation for virus: alfalfa mosaic virus, AMV; pea streak virus, PeSV; lucerne transient streak virus, LTSV; alfalfa dwarf virus, ADV; Medicago sativa alphapartitivirus 1, MsAPV1; Medicago sativa alphapartitivirus 2, MsAPV2; alfalfa leaf curl virus, ALCV; Bean leafroll virus, BLRV; raspberry vein chlorosis virus, RVCV; alfalfa latent virus, ALV; Allium fistulosum carlavirus, AFCLV; Ilex cornuta carlavirus, ICCLV; garlic common latent virus, GCLV; birch carlavirus, BCLV; cowpea mild mottle virus, CPMMV; cherry twisted leaf associated virus, CTLaV; cherry green ring mottle virus, CGRMV; grapevine cabernet sauvignon reovirus, GCSV; raspberry latent virus, RpLV; cassava frogskin virus, CFSV; Medicago sativa amalgavirus 1, MsAGV1 and capsicum chlorosis virus, CaCV. And abbreviation for protein: coat protein, CP; nucleocapsid, N.

Supplementary Table 4. Isolates of alfalfa mosaic virus (AMV) and cucumber mosaic virus (CMV) with complete coat protein nucleotide sequences.

| NO. | Isolate | Accession number | | | host | Geographical origin |
| --- | --- | --- | --- | --- | --- | --- |
|  |  | RNA1 | RNA2 | RNA3 |  |  |
| 1 | China Jiuquan G | OL706243 | OL773498 | OL706255 | *Medicago sativa* | Jiuquan, Gansu, China |
| 2 | China Zhengzhou H1 | OL706244 | OL773499 | OL706256 | *Medicago sativa* | Zhengzhou, Henan, China |
| 3 | China Yuanyang-1 H2 | OL706245 | OL773500 | OL706257 | *Medicago sativa* | Yuanyang, Henan, China |
| 4 | China Yuanyang-2 H3 | OL706246 | OL773501 | OL706258 | *Medicago sativa* | Yuanyang, Henan, China |
| 5 | China Lankao H4 | OL706247 | OL773502 | OL706259 | *Medicago sativa* | Lankao, Henan, China |
| 6 | China Wenxian-1 H5 | OL706248 | OL773503 | OL706260 | *Medicago sativa* | Wenxian, Henan, China |
| 7 | China Wenxian-2 H6 | OL706249 | OL773504 | OL706261 | *Medicago sativa* | Wenxian, Henan, China |
| 8 | China Yichuan H7 | OL706250 | OL773505 | OL706262 | *Medicago sativa* | Yichuan, Henan, China |
| 9 | China Zhenping H8 | OL706251 | OL773506 | OL706263 | *Medicago sativa* | Zhenping, Henan, China |
| 10 | China Helinger N1 | OL706252 | OL773507 | OL706264 | *Medicago sativa* | Helinger, Inner Mongolia, China |
| 11 | China Tumote Left Baaner N2 | OL706253 | OL773508 | OL706265 | *Medicago sativa* | Tumote Left Baaner, Inner Mongolia, China |
| 12 | China Yangling S | OL706254 | OL773509 | OL706266 | *Medicago sativa* | Yangling, Shaanxi, China |
| 13 | China Mint | MK883819 | MK883820 | MK883821 | *Mentha canadensis* | China |
| 14 | USA IA 2017 | MT596806 | MT596807 | MT596808 | *Glycine max* | USA |
| 15 | USA OH 2017 | MT596800 | MT596801 | MT596802 | *Glycine max* | USA |
| 16 | USA MO 2017 | MT596803 | MT596804 | MT596805 | *Glycine max* | USA |
| 17 | USA OH2 2017 | MT669386 | MT669387 | MT669388 | *Glycine max* | USA |
| 18 | USA IA3 2018 | MT596815 | MT596816 | MT596817 | *Glycine max* | USA |
| 19 | Canada CaM | MK607973 | MK607975 | MK607977 | *Solanum tuberosum* | Canada |
| 20 | Argentina Manfredi | KC881008 | KC881009 | KC881010 | *Medicago sativa* | Argentina |
| 21 | China Gyn | MH332897 | MH332898 | MH332899 | *Gynostemma pentaphyllum* | China |
| 22 | Germany DSMZ PV0040 | MZ405629 | MZ405630 | MZ405631 | *Nicotiana tabacum* | Germany |
| 23 | Australia 295 | LC485018 | LC485016 | LC485017 | *Pisum sativum* | Australia |
| 24 | Italy See 1 | MT093209 | MT093210 | MT093211 | *Sechium edule* (Jacq.) | Italy |
| 25 | Australia AU SA80 | MK648424 | MK648425 | MK648426 | *Medicago sativa* | Australia |
| 26 | China HZ | HQ316635 | HQ316636 | HQ316637 | *Nicotiana glutinosa* | China |
| 27 | USA IA4 2018 | MT669391 | MT669392 | MT669393 | *Glycine max* | USA |
| 28 | USA IA1 2018 | MT596809 | MT596810 | MT596811 | *Glycine max* | USA |
| 29 | Germany DSMZ PV0779 | MZ405653 | MZ405654 | MZ405655 | *Nicotiana tabacum* | Germany |
| 30 | Canada Ca175 1 | MK607974 | MK607976 | MK607978 | *Solanum tuberosum* | Canada |
| 31 | Italy Lst | FN667965 | FN667966 | FN667967 | *Lavandula stoechas* | Italy |
| 32 | Canada 175 | MF990284 | MF990285 | MF990286 | *Solanum tuberosum* | Canada |
| 33 | China soybean | MT362607 | MT362608 | MT362609 | *Glycine max* | China |
| 34 | USA IA2 2018 | MT596812 | MT596813 | MT596814 | *Glycine max* | USA |
| 35 | UK FERA160224 | KY810767 | KY810768 | KY810769 | *Nicotiana tabacum* | UK |
| 36 | Spain Tec1 | FR715040 | FR715041 | FR715042 | *Tecomaria capensis* | Spain |
| 37 | Germany DSMZ PV0196 | MZ405636 | MZ405637 | MZ405638 | *Solanum tuberosum* | Germany |
| 38 | China Ib | MT874919 | MT874920 | --- | *Impatiens balsamina* | China |
| 39 | Czech Republic PV1 | MG600287 | --- | MG600289 | *Trifolium pratense* L. | Czech Republic |
| 40 | France Restinclieres 2015 | MW676127 | --- | MW676128 | *Medicago sativa* | France |
| 41 | Egypt ER1 | KX579896 | --- | --- | *Solanum tuberosum* | Egypt |
| 42 | Zambia EP1 | MN326867 | --- | --- | *Phaseolus vulgaris* | Zambia |
| 43 | 425 Leiden | L00163 | --- | --- | *---* | --- |
| 44 | A1M1 | --- | X01572 | --- | *---* | --- |
| 45 | Egypt FER1 | --- | KX463476 | --- | *Solanum tuberosum* | Egypt |
| 46 | China Chinese | --- | DQ139866 | --- | *---* | China |
| 47 | 425 Madison | --- | --- | K02703 | *---* | --- |
| 48 | S | --- | --- | X00819 | *---* | --- |
| 49 | AZ | --- | --- | AB126031 | *---* | --- |
| 50 | Australia N20 | --- | --- | AF332998 | *---* | Australia |
| 51 | Kr | --- | --- | AB126032 | *---* | --- |
| 52 | --- | --- | --- | M59241 | *---* | --- |
| 53 | VRU | --- | --- | AF015716 | *---* | --- |
| 54 | 15/64 | --- | --- | AF015717 | *---* | --- |
| 55 | New Zealand 178 | --- | --- | KC767662 | *Actinidia fortunatii* | New Zealand |
| 56 | --- | --- | --- | K03542 | *---* | --- |
| 57 | New Zealand 176 | --- | --- | KC767661 | *Actinidia glaucophylla* | New Zealand |
| 58 | New Zealand 175 | --- | --- | KC767660 | *Actinidia guilinensis* | New Zealand |
| 59 | Egypt FRE | --- | --- | KY549685 | *---* | Egypt |
| 60 | CMV EP1 | MN326867 | MN326868 | MN326869 | *Phaseolus vulgaris* | Zambia |

Supplementary Table 5. Isolates of pea streak virus (PeSV) and poplar mosaic virus (PopMV) with complete coat protein nucleotide sequences.

| NO. | Isolate | Accession number | Host | Geographical origin |
| --- | --- | --- | --- | --- |
| 1 | China Jiuquan G | MZ221788 | *Medicago sativa* | Jiuquan, Gansu, China |
| 2 | China Helinger N1 | MZ221789 | *Medicago sativa* | Helinger, Inner Mongolia, China |
| 3 | China Tumote Left Baaner N2 | MZ221790 | *Medicago sativa* | Tumote Left Baaner, Inner Mongolia, China |
| 4 | VRS541 | KP828803 | broad bean | --- |
| 5 | Czech Republic V4 | HM107774 | *Medicago sativa* | Czech Republic |
| 6 | USA ATCC PV264 | NC_026616 | *Medicago sativa* | USA |
| 7 | PopMV ATCC PV257 | X65102 | *Populus euramericana* | --- |

Supplementary Table 6. Isolates of lucerne transient streak virus (LTSV) and subterranean clover mottle virus (SCMoV) with complete coat protein nucleotide sequences.

| NO. | Isolate | Accession number | Host | Geographical origin |
| --- | --- | --- | --- | --- |
| 1 | China Helinger N1 | MZ221791 | *Medicago sativa* | Helinger, Inner Mongolia, China |
| 2 | China Tumote Left Baaner N2 | MZ221792 | *Medicago sativa* | Tumote Left Baaner, Inner Mongolia, China |
| 3 | Canada Can | JQ782213 | *Medicago sativa* | Canada |
| 4 | New Zealand | U31286 | --- | New Zealand |
| 5 | USA STN1 | MT224146 | Soybean thrips | USA |
| 6 | SCMoV MJ | AY376453 | --- | Australia |

Supplementary Table 7. Isolates of alfalfa dwarf virus (ADV) and persimmon virus A (PeVA) with complete nucleocapsid nucleotide sequences.

| NO. | Isolate | Accession number | Host | Geographical origin |
| --- | --- | --- | --- | --- |
| 1 | China Jiuquan G | MZ221809 | *Medicago sativa* | Jiuquan, Gansu, China |
| 2 | China Helinger N1 | MZ221810 | *Medicago sativa* | Helinger, Inner Mongolia, China |
| 3 | China Tumote Left Baaner N2 | MZ221811 | *Medicago sativa* | Tumote Left Baaner, Inner Mongolia, China |
| 4 | China Zhengzhou H1 | MZ221812 | *Medicago sativa* | Zhengzhou, Henan, China |
| 5 | China Yuanyang-1 H2 | MZ221813 | *Medicago sativa* | Yuanyang, Henan, China |
| 6 | China Yuanyang-2 H3 | MZ221814 | *Medicago sativa* | Yuanyang, Henan, China |
| 7 | China Wenxian-1 H5 | MZ221815 | *Medicago sativa* | Wenxian, Henan, China |
| 8 | China Wenxian-2 H6 | MZ221816 | *Medicago sativa* | Wenxian, Henan, China |
| 9 | China Won | MH898500 | *Medicago sativa* | China |
| 10 | Argentina Manfredi | KP205452 | *Medicago sativa* | Argentina |
| 11 | PeVA | NC_018381 | *Diospyros kaki* | Japan |

Supplementary Table 8. Isolates of Medicago sativa alphapartitivirus 1 (MsAPV1) and rose partitivirus (RoPV) with complete coat protein nucleotide sequences.

| NO. | Isolate | Accession number | Host | Geographical origin |
| --- | --- | --- | --- | --- |
| 1 | China Jiuquan G | MZ221793 | *Medicago sativa* | Jiuquan, Gansu, China |
| 2 | China Helinger N1 | MZ221794 | *Medicago sativa* | Helinger, Inner Mongolia, China |
| 3 | China Tumote Left Baaner N2 | MZ221795 | *Medicago sativa* | Tumote Left Baaner, Inner Mongolia, China |
| 4 | China Yangling S | MZ221796 | *Medicago sativa* | Yangling, Shaanxi, China |
| 5 | China Zhengzhou H1 | MZ221797 | *Medicago sativa* | Zhengzhou, Henan, China |
| 6 | China Yuanyang-1 H2 | MZ221798 | *Medicago sativa* | Yuanyang, Henan, China |
| 7 | China Yuanyang-2 H3 | MZ221799 | *Medicago sativa* | Yuanyang, Henan, China |
| 8 | China Lankao H4 | MZ221800 | *Medicago sativa* | Lankao, Henan, China |
| 9 | China Wenxian-1 H5 | MZ221801 | *Medicago sativa* | Wenxian, Henan, China |
| 10 | China Wenxian-2 H6 | MZ221802 | *Medicago sativa* | Wenxian, Henan, China |
| 11 | China Yichuan H7 | MZ221803 | *Medicago sativa* | Yichuan, Henan, China |
| 12 | China Zhenping H8 | MZ221804 | *Medicago sativa* | Zhenping, Henan, China |
| 13 | China | MF443257 | *Medicago sativa* | China |
| 14 | USA LN14 | NC_040456 | *Medicago sativa* | USA |
| 15 | USA LN20 | MH846125 | *Medicago sativa* | USA |
| 16 | RoPV PB | KU896859 | *Rosa* sp. cultivar Phyllis Bide | Canada |

Supplementary Table 9. Isolates of Medicago sativa alphapartitivirus 2 (MsAPV2) and rose partitivirus (RoPV) with complete coat protein nucleotide sequences.

| NO. | Isolate | Accession number | Host | Geographical origin |
| --- | --- | --- | --- | --- |
| 1 | China Jiuquan G | MZ221805 | *Medicago sativa* | Jiuquan, Gansu, China |
| 2 | China Tumote Left Baaner N2 | MZ221806 | *Medicago sativa* | Tumote Left Baaner, Inner Mongolia, China |
| 3 | China Yangling S | MZ221807 | *Medicago sativa* | Yangling, Shaanxi, China |
| 4 | China Zhengzhou H1 | MZ221808 | *Medicago sativa* | Zhengzhou, Henan, China |
| 5 | Argentina | MK292289 | *Medicago sativa* | Argentina |
| 6 | RoPV PB | KU896859 | *Rosa* sp. cultivar Phyllis Bide | Canada |

Supplementary Table 10. Isolates of alfalfa leaf curl virus (ALCV) and Euphorbia caput-medusae latent virus (EcmLV) with complete genome nucleotide sequences.

| NO. | Isolate | Accession number | Host | Geographical origin |
| --- | --- | --- | --- | --- |
| 1 | China Helinger N1 | MZ221817 | *Medicago sativa* | Helinger, Inner Mongolia, China |
| 2 | China Tumote Left Baaner N2 | MZ221818 | *Medicago sativa* | Tumote Left Baaner, Inner Mongolia, China |
| 3 | China Zhengzhou H1 | MZ221819 | *Medicago sativa* | Zhengzhou, Henan, China |
| 4 | China Yuanyang-1 H2 | MZ221820 | *Medicago sativa* | Yuanyang, Henan, China |
| 5 | China Yuanyang-2 H3 | MZ221821 | *Medicago sativa* | Yuanyang, Henan, China |
| 6 | China Lankao H4 | MZ221822 | *Medicago sativa* | Lankao, Henan, China |
| 7 | China Wenxian-1 H5 | MZ221823 | *Medicago sativa* | Wenxian, Henan, China |
| 8 | China Wenxian-2 H6 | MZ221824 | *Medicago sativa* | Wenxian, Henan, China |
| 9 | China Yichuan H7 | MZ221825 | *Medicago sativa* | Yichuan, Henan, China |
| 10 | Argentina Manfredi | KX574859 | *Medicago sativa* | Argentina |
| 11 | Argentina Tandil | MG792051 | *Medicago sativa* | Argentina |
| 12 | China SLSC410-1 | MK422438 | *Medicago sativa* | China |
| 13 | France LARAGNE5 | MH603812 | *Medicago sativa* | France |
| 14 | France ENTZ2 | MH603810 | *Medicago sativa* | France |
| 15 | France 44_1E | NC_027714 | *Medicago sativa* | France |
| 16 | Iran Kh17 | MH603829 | *Medicago sativa* | Iran |
| 17 | Iran 254 | MH603827 | *Medicago sativa* | Iran |
| 18 | Italy COURM2 | MH603836 | *Medicago sativa* | Italy |
| 19 | Italy I44 | MH603835 | *Medicago sativa* | Italy |
| 20 | Jordan JoAl31 17 | MH603839 | *Medicago sativa* | Jordan |
| 21 | Lebanon LAl3 17 | MH603841 | *Medicago sativa* | Lebanon |
| 22 | Lebanon Lal2 17 | MH603840 | *Medicago sativa* | Lebanon |
| 23 | Spain ES53 | MH603852 | *Medicago sativa* | Spain |
| 24 | Spain ES52 | MH603851 | *Medicago sativa* | Spain |
| 25 | Syria SyAl45 17 | MH603855 | *Medicago sativa* | Syria |
| 26 | Syria SyAl40 17 | MH603854 | *Medicago sativa* | Syria |
| 27 | Tunisia TuAl20 17 | MH603860 | *Medicago sativa* | Tunisia |
| 28 | Tunisia Tual6 17 | MH020804 | *Medicago sativa* | Tunisia |
| 29 | EcmLV A14 | HF921459 | *Euphorbia caput-medusae* | South Africa |

Supplementary Table 11. Number of illumina reads and contigs per alfalfa sample.

| Samples | Raw reads No. | Clean reads No. | Unique reads No. | No. of contigs | No. of contigs mapped to viruses |
| --- | --- | --- | --- | --- | --- |
| G | 12998499 | 9512942 | 4106060 | 600 | 83 |
| N1 | 11124356 | 7965949 | 2970773 | 372 | 111 |
| N2 | 10575829 | 7889600 | 3060399 | 362 | 58 |
| S | 16082935 | 15668658 | 2140127 | 157 | 57 |
| H1 | 15127108 | 12423321 | 5540407 | 901 | 91 |
| H2 | 14978623 | 11578406 | 5175844 | 709 | 68 |
| H3 | 13276860 | 10436730 | 4249550 | 497 | 40 |
| H4 | 12784277 | 10248917 | 4757769 | 858 | 33 |
| H5 | 10790418 | 8080464 | 3717953 | 634 | 41 |
| H6 | 11300928 | 8284706 | 3636631 | 483 | 39 |
| H7 | 9788810 | 6933449 | 3120666 | 426 | 43 |
| H8 | 10330810 | 8329750 | 3848655 | 537 | 23 |

Supplementary Table 12. Number, total length (bp) and identify of contigs per alfalfa sample aligned with viral reference sequences.

| Samples | No. of contigs | AMV | | | PeSV  8041 | LTSV  4275 | ADV | | MsAPV1 | | MsAPV2 | | ALCV  2750 | BLRV  5964 | RVCV  14667 |
| --- | --- | --- | --- | --- | --- | --- | --- | --- | --- | --- | --- | --- | --- | --- | --- |
|  |  | RNA1  3644 | RNA2  2594 | RNA3  2040 |  |  | Manfredi  14494 | Won  14419 | RdRp  1868 | CP  1806 | RdRp  1939 | CP  1764 |  |  |  |
| G | 83 | 14  3437  ≥97% | 8  2317  ≥91% | 9  1794  ≥97% | 30  7040  ≥90% | --- | 8  864  ≥76% | 7  704  ≥83% | 8  1668≥99% | 4  323  ≥91% | --- | --- | --- | 4  332  ≥77% | 2  190  ≥75% |
| N1 | 111 | 12  3336  ≥88% | 18  2517  ≥88% | 11  1509  ≥89% | 35  5987  ≥77% | 19  2355  ≥93% | 10  1176  ≥72% | 9  1027  ≥89% | 3  368  ≥100% | --- | --- | --- | 9  2185  ≥94% | 2  161  ≥85% | --- |
| N2 | 58 | 10  3565  ≥85% | 8  2354  ≥85% | 9  1669  ≥96% | 16  2041  ≥77% | --- | 3  322  ≥81% | 1  121  ≥92% | --- | --- | --- | --- | 9  2619  ≥96% | 2  216  ≥76% | --- |
| S | 57 | 19  2576  ≥96% | 14  1440  ≥90% | 14  923  ≥94% | --- | --- | --- | --- | 6  529  ≥73% | -- | 5  369  ≥99% | --- | --- | --- | --- |
| H1 | 91 | 5  3492  ≥95% | 7  2366  ≥94% | 8  1813  ≥96% | --- | --- | 5  907  ≥82% | 35  6300  ≥97% | 6  1242  ≥100% | 4  446  ≥98% | --- | --- | 11  2708  ≥96% | --- | --- |
| H2 | 68 | 7  3237  ≥90% | 11  2428  ≥93% | 11  1764  ≥93% | --- | --- | 25  3276  ≥74% | 25  3703  ≥99% | 4  926  ≥99% | 1  141  ≥100% | --- | --- | 11  2586  ≥96% | --- | --- |
| H3 | 40 | 8  3341  ≥95% | 11  2366  ≥88% | 8  1739  ≥93% | --- | --- | --- | --- | 2  296  ≥100% | 1  42  ≥100% | --- | 1  42  ≥100% | 12  2546  ≥98% | --- | --- |
| H4 | 33 | 8  3542  ≥92% | 8  2367  ≥85% | 6  1677  ≥94% | --- | --- | --- | --- | 3  501  ≥99% | 1  86  ≥100% | --- | --- | 9  2630  ≥98% | --- | --- |
| H5 | 41 | 11  3171  ≥96% | 8  1851  ≥97% | 5  1667  ≥97% | --- | --- | 5  626  ≥75% | 4  485  ≥99% | 5  801  ≥100% | --- | --- | --- | 7  2577  ≥98% | --- | --- |
| H6 | 39 | 12  3237  ≥95% | 9  2270  ≥95% | 11  1463  ≥96% | --- | --- | --- | --- | --- | --- | --- | --- | 10  2534  ≥99% | --- | --- |
| H7 | 43 | 12  3176  ≥97% | 8  2328  ≥93% | 12  1941  ≥96% | --- | --- | --- | --- | 3  346  ≥100% | --- | --- | --- | 11  2572  ≥98% | --- | --- |
| H8 | 23 | 10  3548  ≥90% | 6  2433  ≥90% | 5  1738  ≥94% | --- | --- | --- | --- | 3  372  ≥100% | --- | --- | --- | --- | --- | --- |

Continued Table 12.

| Samples | No. of contigs | ALV  8041 | AFCLV  8260 | ICCLV  8344 | GCLV  8638 | BCLV  8896 | CPMMV  8189 | CTLaV  8426 | CGRMV  8353 | GCSV  24596 | RpLV  26128 | CFSV Segment 4  1464 | MsAGV1  3423 | CaCV  Segment S  3944 |
| --- | --- | --- | --- | --- | --- | --- | --- | --- | --- | --- | --- | --- | --- | --- |
| G | 83 | --- | --- | --- | --- | --- | --- | --- | --- | --- | --- | --- | --- | --- |
| N1 | 111 | 34  5821  ≥71% | 3  373  ≥71% | 4  411  ≥73% | 4  441  ≥68% | 3  281  ≥72% | 5  473  ≥71% | 1  134  ≥76% | 3  194  ≥72% | --- | --- | --- | --- | --- |
| N2 | 58 | 19  2541  ≥71% | --- | --- | --- | --- | --- | --- | --- | --- | --- | --- | --- | --- |
| S | 44 | --- | --- | --- | --- | --- | --- | --- | --- | --- | --- | --- | --- | --- |
| H1 | 91 | --- | --- | --- | --- | --- | --- | --- | --- | 12  1234  ≥70% | 4  351  ≥72% | 1  104  ≥79% | --- | --- |
| H2 | 68 | --- | --- | --- | --- | --- | --- | --- | --- | --- | --- | --- | --- | --- |
| H3 | 40 | --- | --- | --- | --- | --- | --- | --- | --- | --- | --- | --- | 1  31  ≥100% | --- |
| H4 | 33 | --- | --- | --- | --- | --- | --- | --- | --- | --- | --- | --- | --- | --- |
| H5 | 41 | --- | --- | --- | --- | --- | --- | --- | --- | --- | --- | --- | --- | --- |
| H6 | 39 | --- | --- | --- | --- | --- | --- | --- | --- | --- | --- | --- | --- | --- |
| H7 | 43 | --- | --- | --- | --- | --- | --- | --- | --- | --- | --- | --- | --- | --- |
| H8 | 23 | --- | --- | --- | --- | --- | --- | --- | --- | --- | --- | --- | --- | 3  409  ≥97% |

Note: The accession numbers of virus sequences used for reference mapping: AMV RNA 1-3: MK883819, MK883820 and MK883821, PeSV: KP828803, LTSV: U31286, ADV Manfredi: NC_028237, ADV Won: MH898500, MsAPV1-RdRp: MH846126, MsAPV1-CP: MK292287, MsAPV2-RdRp: MK292288, MsAPV2-CP: MK292289, ALCV: MK422438, BLRV: AF441393, RVCV: MK257717, ALV: KP784454, AFCLV: MN814318, ICCLV: MN814320, GCLV: JF320810, BCLV: MH536506, CMMV: MH345698, CTLaV: KF030846, CGRMV: LC522990, GCSV: NC_027802, NC_027808, NC_027809, NC_027810, NC_027816, NC_035935, NC_035936, NC_035937, NC_035938 and NC_035939, RpLV S1-10: NC_014598, NC_014599, NC_014600, NC_014601, NC_014602, NC_014603, NC_014604, NC_014605, NC_014606 and NC_014607, CsFSV Segment 4: AH015299, MsAGV1: MK648427, CaCV segment S: KM589495.

Supplementary Table 13. Virus incidence of collected samples from 12 locations in four provinces of China.

| Field location | | | | | | | | | | | | |
| --- | --- | --- | --- | --- | --- | --- | --- | --- | --- | --- | --- | --- |
|  | G | N1 | N2 | S | H1 | H2 | H3 | H4 | H5 | H6 | H7 | H8 |
| Incidence | 84.50% | 80.00% | 89.83% | 65.00% | 100% | 100% | 100% | 100% | 100% | 100% | 100% | 100% |

Supplementary Table 14. Numbers of samples infected with single and multiple viruses in alfalfa in Jiuquan of Gansu provinces (G) of China.

| Virus | Total samples | Single infection | Dual infections | | | | | | | Multiple infections | | | | | | | |
| --- | --- | --- | --- | --- | --- | --- | --- | --- | --- | --- | --- | --- | --- | --- | --- | --- | --- |
|  |  |  | AMV | PeSV | LTSV | ADV | MsAPV1 | MsAPV2 | ALCV |  | AMV | PeSV | LTSV | ADV | MsAPV1 | MsAPV2 | ALCV |
| AMV | 258 | 14 | --- | 44 | 0 | 0 | 6 | 0 | 0 | AMV+PeSV+ADV | 2 | 2 | 0 | 2 | 0 | 0 | 0 |
| PeSV | 258 | 12 | 44 | --- | 0 | 2 | 0 | 0 | 0 | AMV+PeSV+MsAPV1 | 108 | 108 | 0 | 0 | 108 | 0 | 0 |
| LTSV | 258 | 0 | 0 | 0 | --- | 0 | 0 | 0 | 0 | AMV+PeSV+MsAPV2 | 12 | 12 | 0 | 0 | 0 | 12 | 0 |
| ADV | 258 | 0 | 0 | 2 | 0 | --- | 0 | 0 | 0 | AMV+PeSV+  ADV+MsAPV1 | 10 | 10 | 0 | 10 | 10 | 0 | 0 |
| MsAPV1 | 258 | 0 | 6 | 0 | 0 | 0 | --- | 0 | 0 | AMV+PeSV+  MsAPV1+MsAPV2 | 6 | 6 | 0 | 0 | 6 | 6 | 0 |
| MsAPV2 | 258 | 0 | 0 | 0 | 0 | 0 | 0 | --- | 0 | AMV+PeSV+ADV +MsAPV1+MsAPV2 | 2 | 2 | 0 | 2 | 2 | 2 | 0 |
| ALCV | 258 | 0 | 0 | 0 | 0 | 0 | 0 | 0 | --- |  |  |  |  |  |  |  |  |
| Total |  | 26 | 50 | 46 | 0 | 2 | 6 | 0 | 0 |  | 140 | 140 | 0 | 14 | 126 | 20 | 0 |

Supplementary Table 15. Numbers of samples infected with single and multiple viruses in alfalfa in Helinger of Inner Mongolia Autonomous Region (N1) of China.

| Virus | Total samples | Single infection | Dual infections | | | | | | | Multiple infections | | | | | | | |
| --- | --- | --- | --- | --- | --- | --- | --- | --- | --- | --- | --- | --- | --- | --- | --- | --- | --- |
|  |  |  | AMV | PeSV | LTSV | ADV | MsAPV1 | MsAPV2 | ALCV |  | AMV | PeSV | LTSV | ADV | MsAPV1 | MsAPV2 | ALCV |
| AMV | 80 | 10 | --- | 16 | 4 | 2 | 12 | 0 | 0 | AMV+PeSV+ADV | 4 | 4 | 0 | 4 | 0 | 0 | 0 |
| PeSV | 80 | 2 | 16 | --- | 0 | 0 | 0 | 0 | 0 | AMV+PeSV+ALCV | 4 | 4 | 0 | 0 | 0 | 0 | 4 |
| LTSV | 80 | 0 | 4 | 0 | --- | 0 | 0 | 0 | 0 | AMV+LTSV+MsAPV1 | 2 | 0 | 2 | 0 | 2 | 0 | 0 |
| ADV | 80 | 0 | 2 | 0 | 0 | --- | 0 | 0 | 0 | AMV+ADV+MsAPV1 | 2 | 0 | 0 | 2 | 2 | 0 | 0 |
| MsAPV1 | 80 | 0 | 12 | 0 | 0 | 0 | --- | 0 | 0 | AMV+PeSV+  ADV+MsAPV1 | 4 | 4 | 0 | 4 | 4 | 0 | 0 |
| MsAPV2 | 80 | 0 | 0 | 0 | 0 | 0 | 0 | --- | 0 | AMV+PeSV+  ADV+ALCV | 2 | 2 | 0 | 2 | 0 | 0 | 2 |
| ALCV | 80 | 0 | 0 | 0 | 0 | 0 | 0 | 0 | --- |  |  |  |  |  |  |  |  |
| Total |  | 12 | 34 | 16 | 4 | 2 | 12 | 0 | 0 |  | 18 | 14 | 2 | 12 | 8 | 0 | 6 |

Supplementary Table 16. Numbers of samples infected with single and multiple viruses in alfalfa in Tumote Left Banner of Inner Mongolia Autonomous Region (N2) of China.

| Virus | Total samples | Single infection | Dual infections | | | | | | | Multiple infections | | | | | | | |
| --- | --- | --- | --- | --- | --- | --- | --- | --- | --- | --- | --- | --- | --- | --- | --- | --- | --- |
|  |  |  | AMV | PeSV | LTSV | ADV | MsAPV1 | MsAPV2 | ALCV |  | AMV | PeSV | LTSV | ADV | MsAPV1 | MsAPV2 | ALCV |
| AMV | 118 | 12 | --- | 4 | 0 | 6 | 0 | 2 | 10 | AMV+PeSV+ADV | 6 | 6 | 0 | 6 | 0 | 0 | 0 |
| PeSV | 118 | 2 | 4 | --- | 0 | 2 | 0 | 4 | 0 | AMV+PeSV+MsAPV1 | 2 | 2 | 0 | 0 | 2 | 0 | 0 |
| LTSV | 118 | 0 | 0 | 0 | --- | 0 | 0 | 0 | 0 | AMV+PeSV+MsAPV2 | 2 | 2 | 0 | 0 | 0 | 2 | 0 |
| ADV | 118 | 2 | 6 | 2 | 0 | --- | 0 | 0 | 0 | AMV+PeSV+ALCV | 4 | 4 | 0 | 0 | 0 | 0 | 4 |
| MsAPV1 | 118 | 2 | 0 | 0 | 0 | 0 | --- | 0 | 0 | AMV+ADV+MsAPV2 | 2 | 0 | 0 | 2 | 0 | 2 | 0 |
| MsAPV2 | 118 | 0 | 2 | 4 | 0 | 0 | 0 | --- | 0 | AMV+ADV+ALCV | 4 | 0 | 0 | 4 | 0 | 0 | 4 |
| ALCV | 118 | 6 | 10 | 0 | 0 | 0 | 0 | 0 | --- | AMV+MsAPV1+ALCV | 2 | 0 | 0 | 0 | 2 | 0 | 2 |
|  |  |  |  |  |  |  |  |  |  | AMV+MsAPV2+ALCV | 2 | 0 | 0 | 0 | 0 | 2 | 2 |
|  |  |  |  |  |  |  |  |  |  | PeSV+ADV+ALCV | 0 | 4 | 4 | 0 | 0 | 0 | 4 |
|  |  |  |  |  |  |  |  |  |  | PeSV+MsAPV1+ALCV | 0 | 2 | 0 | 0 | 2 | 0 | 2 |
|  |  |  |  |  |  |  |  |  |  | ADV+MsAPV2+ALCV | 0 | 0 | 0 | 2 | 0 | 2 | 2 |
|  |  |  |  |  |  |  |  |  |  | AMV+PeSV+  ADV+MsAPV1 | 2 | 2 | 0 | 2 | 2 | 0 | 0 |
|  |  |  |  |  |  |  |  |  |  | AMV+PeSV+  ADV+MsAPV2 | 4 | 4 | 0 | 4 | 0 | 4 | 0 |
|  |  |  |  |  |  |  |  |  |  | AMV+PeSV+  ADV+ALCV | 2 | 2 | 0 | 2 | 0 | 0 | 2 |
|  |  |  |  |  |  |  |  |  |  | AMV+ADV +MsAPV1+MsAPV2 | 2 | 0 | 0 | 2 | 2 | 2 | 0 |
|  |  |  |  |  |  |  |  |  |  | AMV+MsAPV1+  MsAPV2+ALCV | 2 | 0 | 0 | 0 | 2 | 2 | 2 |
|  |  |  |  |  |  |  |  |  |  | PeSV+ADV +MsAPV1+ALCV | 0 | 2 | 0 | 2 | 2 | 0 | 2 |
|  |  |  |  |  |  |  |  |  |  | AMV+PeSV+LTSV+  ADV+MsAPV1 | 2 | 2 | 2 | 2 | 2 | 0 | 0 |
|  |  |  |  |  |  |  |  |  |  | AMV+PeSV+ADV +MsAPV1+ALCV | 2 | 2 | 0 | 2 | 2 | 0 | 2 |
|  |  |  |  |  |  |  |  |  |  | AMV+PeSV+ADV +MsAPV2+ALCV | 4 | 4 | 0 | 4 | 0 | 4 | 4 |
| Total |  | 24 | 22 | 10 | 0 | 8 | 0 | 6 | 10 |  | 44 | 38 | 6 | 34 | 18 | 20 | 32 |

Supplementary Table 17. Numbers of samples infected with single and multiple viruses in alfalfa in Yangling of Shaanxi province (S) of China.

| Virus | Total samples | Single infection | Dual infections | | | | | | | Multiple infections | | | | | | | |
| --- | --- | --- | --- | --- | --- | --- | --- | --- | --- | --- | --- | --- | --- | --- | --- | --- | --- |
|  |  |  | AMV | PeSV | LTSV | ADV | MsAPV1 | MsAPV2 | ALCV |  | AMV | PeSV | LTSV | ADV | MsAPV1 | MsAPV2 | ALCV |
| AMV | 80 | 6 | --- | 0 | 0 | 0 | 32 | 0 | 0 | AMV+MsAPV1+MsAPV2 | 8 | 0 | 0 | 0 | 8 | 8 | 0 |
| PeSV | 80 | 0 | 0 | --- | 0 | 0 | 0 | 0 | 0 |  |  |  |  |  |  |  |  |
| LTSV | 80 | 0 | 0 | 0 | --- | 0 | 0 | 0 | 0 |  |  |  |  |  |  |  |  |
| ADV | 80 | 0 | 0 | 0 | 0 | --- | 0 | 0 | 0 |  |  |  |  |  |  |  |  |
| MsAPV1 | 80 | 6 | 32 | 0 | 0 | 0 | --- | 0 | 0 |  |  |  |  |  |  |  |  |
| MsAPV2 | 80 | 0 | 0 | 0 | 0 | 0 | 0 | --- | 0 |  |  |  |  |  |  |  |  |
| ALCV | 80 | 0 | 0 | 0 | 0 | 0 | 0 | 0 | --- |  |  |  |  |  |  |  |  |
| Total |  | 12 | 32 | 0 | 0 | 0 | 32 | 0 | 0 |  | 8 | 0 | 0 | 0 | 8 | 8 | 0 |

Supplementary Table 18. Numbers of samples infected with single and multiple viruses in alfalfa in Zhengzhou of Henan province (H1) of China.

| Virus | Total samples | Single infection | Dual infections | | | | | | | Multiple infections | | | | | | | |
| --- | --- | --- | --- | --- | --- | --- | --- | --- | --- | --- | --- | --- | --- | --- | --- | --- | --- |
|  |  |  | AMV | PeSV | LTSV | ADV | MsAPV1 | MsAPV2 | ALCV |  | AMV | PeSV | LTSV | ADV | MsAPV1 | MsAPV2 | ALCV |
| AMV | 120 | 4 | --- | 0 | 0 | 0 | 34 | 0 | 2 | AMV+ADV+MsAPV1 | 48 | 0 | 0 | 48 | 48 | 0 | 0 |
| PeSV | 120 | 0 | 0 | --- | 0 | 0 | 0 | 0 | 0 | AMV+MsAPV1+MsAPV2 | 8 | 0 | 0 | 0 | 8 | 8 | 0 |
| LTSV | 120 | 0 | 0 | 0 | --- | 0 | 0 | 0 | 0 | AMV+MsAPV1+ALCV | 4 | 0 | 0 | 0 | 8 | 0 | 8 |
| ADV | 120 | 0 | 0 | 0 | 0 | --- | 2 | 0 | 0 | AMV+ADV +MsAPV1+MsAPV2 | 4 | 0 | 0 | 4 | 4 | 4 | 0 |
| MsAPV1 | 120 | 8 | 34 | 0 | 0 | 2 | --- | 0 | 0 | AMV+ADV +MsAPV1+ALCV | 6 | 0 | 0 | 6 | 6 | 0 | 6 |
| MsAPV2 | 120 | 0 | 0 | 0 | 0 | 0 | 0 | --- | 0 |  | 0 | 0 | 0 | 0 | 0 | 0 | 0 |
| ALCV | 120 | 0 | 2 | 0 | 0 | 0 | 0 | 0 | --- |  | 0 | 0 | 0 | 0 | 0 | 0 | 0 |
| Total |  | 12 | 36 | 0 | 0 | 2 | 36 | 0 | 2 |  | 70 | 0 | 0 | 58 | 74 | 12 | 14 |

Supplementary Table 19. Numbers of samples infected with single and multiple viruses in alfalfa in Yuanyang-1 of Henan province (H2) of China.

| Virus | Total samples | Single infection | Dual infections | | | | | | | | Multiple infections | | | | | | | |
| --- | --- | --- | --- | --- | --- | --- | --- | --- | --- | --- | --- | --- | --- | --- | --- | --- | --- | --- |
|  |  |  | AMV | PeSV | LTSV | ADV | MsAPV1 | MsAPV2 | ALCV |  | | AMV | PeSV | LTSV | ADV | MsAPV1 | MsAPV2 | ALCV |
| AMV | 108 | 14 | --- | 0 | 0 | 4 | 36 | 0 | 0 | AMV+ADV+MsAPV1 | | 32 | 0 | 0 | 32 | 32 | 0 | 0 |
| PeSV | 108 | 0 | 0 | --- | 0 | 0 | 0 | 0 | 0 | AMV+MsAPV1+ALCV | | 8 | 0 | 0 | 0 | 8 | 0 | 8 |
| LTSV | 108 | 0 | 0 | 0 | --- | 0 | 0 | 0 | 0 | AMV+ADV +MsAPV1+ALCV | | 6 | 0 | 0 | 6 | 6 | 0 | 6 |
| ADV | 108 | 2 | 4 | 0 | 0 | --- | 4 | 0 | 0 |  | |  |  |  |  |  |  |  |
| MsAPV1 | 108 | 0 | 36 | 0 | 0 | 4 | --- | 0 | 2 |  | |  |  |  |  |  |  |  |
| MsAPV2 | 108 | 0 | 0 | 0 | 0 | 0 | 0 | --- | 0 |  | |  |  |  |  |  |  |  |
| ALCV | 108 | 0 | 0 | 0 | 0 | 0 | 2 | 0 | --- |  | |  |  |  |  |  |  |  |
| Total |  | 16 | 40 | 0 | 0 | 8 | 42 | 0 | 2 |  | | 46 | 0 | 0 | 38 | 46 | 0 | 14 |

Supplementary Table 20. Numbers of samples infected with single and multiple viruses in alfalfa in Yuanyang-2 of Henan province (H3) of China.

| Virus | Total samples | Single infection | Dual infections | | | | | | | Multiple infections | | | | | | | |
| --- | --- | --- | --- | --- | --- | --- | --- | --- | --- | --- | --- | --- | --- | --- | --- | --- | --- |
|  |  |  | AMV | PeSV | LTSV | ADV | MsAPV1 | MsAPV2 | ALCV |  | AMV | PeSV | LTSV | ADV | MsAPV1 | MsAPV2 | ALCV |
| AMV | 40 | 6 | --- | 0 | 0 | 0 | 20 | 0 | 2 | AMV+MsAPV1+ALCV | 8 | 0 | 0 | 0 | 8 | 0 | 8 |
| PeSV | 40 | 0 | 0 | --- | 0 | 0 | 0 | 0 | 0 | AMV+ADV +MsAPV1+ALCV | 4 | 0 | 0 | 4 | 4 | 0 | 4 |
| LTSV | 40 | 0 | 0 | 0 | --- | 0 | 0 | 0 | 0 |  |  |  |  |  |  |  |  |
| ADV | 40 | 0 | 0 | 0 | 0 | --- | 0 | 0 | 0 |  |  |  |  |  |  |  |  |
| MsAPV1 | 40 | 0 | 20 | 0 | 0 | 0 | --- | 0 | 0 |  |  |  |  |  |  |  |  |
| MsAPV2 | 40 | 0 | 0 | 0 | 0 | 0 | 0 | --- | 0 |  |  |  |  |  |  |  |  |
| ALCV | 40 | 0 | 2 | 0 | 0 | 0 | 0 | 0 | --- |  |  |  |  |  |  |  |  |
| Total |  | 6 | 22 | 0 | 0 | 0 | 20 | 0 | 2 |  | 12 | 0 | 0 | 4 | 12 | 0 | 12 |

Supplementary Table 21. Numbers of samples infected with single and multiple viruses in alfalfa in Lankao of Henan province (H4) of China.

| Virus | Total samples | Single infection | Dual infections | | | | | | | | Multiple infections | | | | | | | |
| --- | --- | --- | --- | --- | --- | --- | --- | --- | --- | --- | --- | --- | --- | --- | --- | --- | --- | --- |
|  |  |  | AMV | PeSV | LTSV | ADV | MsAPV1 | MsAPV2 | ALCV |  | | AMV | PeSV | LTSV | ADV | MsAPV1 | MsAPV2 | ALCV |
| AMV | 60 | 6 | --- | 0 | 0 | 0 | 50 | 0 | 0 | AMV+MsAPV1+ALCV | | 4 | 0 | 0 | 0 | 4 | 0 | 4 |
| PeSV | 60 | 0 | 0 | --- | 0 | 0 | 0 | 0 | 0 |  | |  |  |  |  |  |  |  |
| LTSV | 60 | 0 | 0 | 0 | --- | 0 | 0 | 0 | 0 |  | |  |  |  |  |  |  |  |
| ADV | 60 | 0 | 0 | 0 | 0 | --- | 0 | 0 | 0 |  | |  |  |  |  |  |  |  |
| MsAPV1 | 60 | 0 | 50 | 0 | 0 | 0 | --- | 0 | 0 |  | |  |  |  |  |  |  |  |
| MsAPV2 | 60 | 0 | 0 | 0 | 0 | 0 | 0 | --- | 0 |  | |  |  |  |  |  |  |  |
| ALCV | 60 | 0 | 0 | 0 | 0 | 0 | 0 | 0 | --- |  | |  |  |  |  |  |  |  |
| Total |  | 6 | 50 | 0 | 0 | 0 | 50 | 0 | 0 |  | | 4 | 0 | 0 | 0 | 4 | 0 | 4 |

Supplementary Table 22. Numbers of samples infected with single and multiple viruses in alfalfa in Wenxian-1 of Henan province (H5) of China.

| Virus | Total samples | Single infection | Dual infections | | | | | | | Multiple infections | | | | | | | |
| --- | --- | --- | --- | --- | --- | --- | --- | --- | --- | --- | --- | --- | --- | --- | --- | --- | --- |
|  |  |  | AMV | PeSV | LTSV | ADV | MsAPV1 | MsAPV2 | ALCV |  | AMV | PeSV | LTSV | ADV | MsAPV1 | MsAPV2 | ALCV |
| AMV | 44 | 0 | --- | 0 | 0 | 0 | 2 | 0 | 0 | AMV+MsAPV1+ALCV | 6 | 0 | 0 | 0 | 6 | 0 | 6 |
| PeSV | 44 | 0 | 0 | --- | 0 | 0 | 0 | 0 | 0 |  |  |  |  |  |  |  |  |
| LTSV | 44 | 0 | 0 | 0 | --- | 0 | 0 | 0 | 0 |  |  |  |  |  |  |  |  |
| ADV | 44 | 0 | 0 | 0 | 0 | --- | 6 | 0 | 0 |  |  |  |  |  |  |  |  |
| MsAPV1 | 44 | 26 | 2 | 0 | 0 | 6 | --- | 0 | 4 |  |  |  |  |  |  |  |  |
| MsAPV2 | 44 | 0 | 0 | 0 | 0 | 0 | 0 | --- | 0 |  |  |  |  |  |  |  |  |
| ALCV | 44 | 0 | 0 | 0 | 0 | 0 | 4 | 0 | --- |  |  |  |  |  |  |  |  |
| Total |  | 26 | 2 | 0 | 0 | 6 | 12 | 0 | 4 |  | 6 | 0 | 0 | 0 | 6 | 0 | 6 |

Supplementary Table 23. Numbers of samples infected with single and multiple viruses in alfalfa in Wenxian-2 of Henan province (H6) of China.

| Virus | Total samples | Single infection | Dual infections | | | | | | | Multiple infections | | | | | | | |
| --- | --- | --- | --- | --- | --- | --- | --- | --- | --- | --- | --- | --- | --- | --- | --- | --- | --- |
|  |  |  | AMV | PeSV | LTSV | ADV | MsAPV1 | MsAPV2 | ALCV |  | AMV | PeSV | LTSV | ADV | MsAPV1 | MsAPV2 | ALCV |
| AMV | 40 | 2 | --- | 0 | 0 | 2 | 6 | 0 | 2 | AMV+MsAPV1+ALCV | 22 | 0 | 0 | 0 | 22 | 0 | 22 |
| PeSV | 40 | 0 | 0 | --- | 0 | 0 | 0 | 0 | 0 | ADV+MsAPV1+ALCV | 0 | 0 | 0 | 2 | 2 | 0 | 2 |
| LTSV | 40 | 0 | 0 | 0 | --- | 0 | 0 | 0 | 0 | AMV+ADV +MsAPV1+ALCV | 2 | 0 | 0 | 2 | 2 | 0 | 2 |
| ADV | 40 | 0 | 2 | 0 | 0 | --- | 0 | 0 | 0 |  |  |  |  |  |  |  |  |
| MsAPV1 | 40 | 0 | 6 | 0 | 0 | 0 | --- | 0 | 2 |  |  |  |  |  |  |  |  |
| MsAPV2 | 40 | 0 | 0 | 0 | 0 | 0 | 0 | --- | 0 |  |  |  |  |  |  |  |  |
| ALCV | 40 | 0 | 2 | 0 | 0 | 0 | 2 | 0 | --- |  |  |  |  |  |  |  |  |
| Total |  | 2 | 10 | 0 | 0 | 2 | 8 | 0 | 4 |  | 24 | 0 | 0 | 4 | 26 | 0 | 26 |

Supplementary Table 24. Numbers of samples infected with single and multiple viruses in alfalfa in Yichuan of Henan province (H7) of China.

| Virus | Total samples | Single infection | Dual infections | | | | | | | | Multiple infections | | | | | | | |
| --- | --- | --- | --- | --- | --- | --- | --- | --- | --- | --- | --- | --- | --- | --- | --- | --- | --- | --- |
|  |  |  | AMV | PeSV | LTSV | ADV | MsAPV1 | MsAPV2 | ALCV |  | | AMV | PeSV | LTSV | ADV | MsAPV1 | MsAPV2 | ALCV |
| AMV | 60 | 2 | --- | 0 | 0 | 0 | 24 | 0 | 2 | AMV+MsAPV1+ALCV | | 28 | 0 | 0 | 0 | 28 | 0 | 28 |
| PeSV | 60 | 0 | 0 | --- | 0 | 0 | 0 | 0 | 0 |  | |  |  |  |  |  |  |  |
| LTSV | 60 | 0 | 0 | 0 | --- | 0 | 0 | 0 | 0 |  | |  |  |  |  |  |  |  |
| ADV | 60 | 0 | 0 | 0 | 0 | --- | 0 | 0 | 0 |  | |  |  |  |  |  |  |  |
| MsAPV1 | 60 | 2 | 24 | 0 | 0 | 0 | --- | 0 | 2 |  | |  |  |  |  |  |  |  |
| MsAPV2 | 60 | 0 | 0 | 0 | 0 | 0 | 0 | --- | 0 |  | |  |  |  |  |  |  |  |
| ALCV | 60 | 0 | 2 | 0 | 0 | 0 | 2 | 0 | --- |  | |  |  |  |  |  |  |  |
| Total |  | 4 | 26 | 0 | 0 | 0 | 26 | 0 | 4 |  | | 28 | 0 | 0 | 0 | 28 | 0 | 28 |

Supplementary Table 25. Numbers of samples infected with single and multiple viruses in alfalfa in Zhenping of Henan province (H8) of China.

| Virus | Total samples | Single infection | Dual infections | | | | | | | Multiple infections | | | | | | | |
| --- | --- | --- | --- | --- | --- | --- | --- | --- | --- | --- | --- | --- | --- | --- | --- | --- | --- |
|  |  |  | AMV | PeSV | LTSV | ADV | MsAPV1 | MsAPV2 | ALCV |  | AMV | PeSV | LTSV | ADV | MsAPV1 | MsAPV2 | ALCV |
| AMV | 60 | 6 | --- | 0 | 0 | 0 | 48 | 0 | 0 | --- | --- | --- | --- | --- | --- | --- | --- |
| PeSV | 60 | 0 | 0 | --- | 0 | 0 | 0 | 0 | 0 |  |  |  |  |  |  |  |  |
| LTSV | 60 | 0 | 0 | 0 | --- | 0 | 0 | 0 | 0 |  |  |  |  |  |  |  |  |
| ADV | 60 | 0 | 0 | 0 | 0 | --- | 0 | 0 | 0 |  |  |  |  |  |  |  |  |
| MsAPV1 | 60 | 6 | 48 | 0 | 0 | 0 | --- | 0 | 0 |  |  |  |  |  |  |  |  |
| MsAPV2 | 60 | 0 | 0 | 0 | 0 | 0 | 0 | --- | 0 |  |  |  |  |  |  |  |  |
| ALCV | 60 | 0 | 0 | 0 | 0 | 0 | 0 | 0 | --- |  |  |  |  |  |  |  |  |
| Total |  | 12 | 48 | 0 | 0 | 0 | 48 | 0 | 0 |  | 0 | 0 | 0 | 0 | 0 | 0 | 0 |
